# Diversity of *Bacillus cereus* sensu *lato* mobilome

**DOI:** 10.1186/s12864-019-5764-4

**Published:** 2019-05-29

**Authors:** Nancy Fayad, Mireille Kallassy Awad, Jacques Mahillon

**Affiliations:** 1 Laboratory of Food and Environmental Microbiology, Earth and Life Institute, UCLouvain, Croix du Sud, 2 - L7.05.12, B-1348 Louvain-la-Neuve, Belgium; 2 Laboratory of Biodiversity and Functional Genomics, Faculty of Science, Université Saint-Joseph de Beyrouth, Beirut, Lebanon

**Keywords:** *Bacillus anthracis*, *Bacillus cereus* repeats, *Bacillus thuringiensis*, Group II introns, Insertion sequences, Transposons

## Abstract

**Background:**

*Bacillus cereus sensu lato s.l.*) is a group of bacteria displaying close phylogenetic relationships but a high ecological diversity. The three most studied species are *Bacillus anthracis, Bacillus cereus sensu stricto* and *Bacillus thuringiensis*. While some species are pathogenic to mammals or associated with food poisoning, *Bacillus thuringiensis* is a well-known entomopathogenic bacterium used as biopesticide worldwide. *B. cereus s.l.* also contains a large variety of mobile genetic elements (MGEs).

**Results:**

In this study, we detail the occurrence and plasmid vs. chromosome distribution of several MGEs in 102 complete and annotated genomes of *B. cereus s.l.* These MGEs include 16 Insertion Sequence (IS) families, the Tn*3* family, 18 different *Bacillus cereus* repeats (*BCRs*) and 30 known group II introns.

**Conclusions:**

Our analysis not only shows the diversity of these MGEs among strains of the same species and between different species within the *B. cereus s.l.* group, but also highlights the potential impact of these elements on the plasticity of the plasmid pool, and the TEs (Transposable Elements) - species relationship within *B. cereus s.l.*

**Electronic supplementary material:**

The online version of this article (10.1186/s12864-019-5764-4) contains supplementary material, which is available to authorized users.

## Background

*Bacillus cereus* group, also known as *B. cereus sensu lato s.l.*), is a group of Gram-positive spore-forming bacteria that comprises nine ecologically diverse species with a wide pathogenicity spectrum [1], despite being phylogenetically close. The most studied species of this group are *B. anthracis,* the anthrax causative pathogen [2, 3], *B. cereus sensu stricto (s.s.)*, to which belong foodborne pathogenic strains, and *B. thuringiensis,* an entomopathogenic bacterium widely used as biopesticide [4, 5]. *B. thuringiensis* insecticidal toxins, also known as δ-endotoxins, form a parasporal crystal, released in the environment at the end of the sporulation phase. The six other species are *Bacillus weihenstephanensis* [6]*, Bacillus cytotoxicus* [7]*, Bacillus mycoides, Bacillus pseudomycoides* [8]*, Bacillus toyonensis* [9] and *Bacillus cereus* biovar *anthracis* [10]. Recently, *Bacillus gaemokensis* [11]*, Bacillus manliponensis* [12], *Bacillus bingmayongensis* [13] and *Bacillus wiedmannii* [14] have also been suggested as distinct species of this group.

Over the years, the phylogeny of *B. cereus s.l.* has been extensively analysed*.* In fact, classification of the species within this group is crucial given the intensive use of *B. thuringiensis* in the field. However, the species delineation between *B. thuringiensis* and *B. cereus s.s.* within the *B. cereus* group has been problematic despite the various approaches and techniques used. The efforts to distinguish between these two species have used phenotypical observations [15], 16S rRNA and housekeeping genes analysis [16] and whole-genome based approaches. The *B. cereus s.l.* pan-genome consists of about 60,000 genes, with 600 core-genes. Using this pan-genome and several phylogenetic approaches, followed by a Bayesian model, population genetic analysis revealed that *B. cereus s.l.* is mainly divided into three clades [17]. Similarly to other studies though, either based on MLST (Multi Locus Sequence Typing), maximum-likelihood phylogeny or peptidome analysis, the *B. thuringiensis* and *B. cereus* distinction within the three clades could not be resolved [17–20]. Another study however was able to make the distinction between *B. cereus s.s.*, *B. thuringiensis* and *B. anthracis* by combining a digital DNA-DNA hybridization technique, 16S rRNA and housekeeping gene analysis, a novel MLST approach and screening of toxin-coding genes [21]. This study divided 224 analysed *B. cereus s.l.* genomes into 30 groups, each representing independent species with 19 new ones. Yet another study was also able to differentiate *B. cereus s.l.* species on a plasmidial level using Genome Target Evaluator (GTEvaluator) and several genetic markers [22]. In fact it was suggested that plasmids, especially large toxin-carrying plasmids, play a crucial role in the phenotypical heterogeneity of the *B. cereus* group [23]. Not only do *B. thuringiensis* strains possess variable plasmid numbers, but also these plasmids are very versatile. They may carry the delta-endotoxin coding genes, such as pBtoxis in *B. thuringiensis* sv. (serovar) *israelensis* [24, 25], and some are conjugative (e.g. pXO16, [26] and pAW63, [27]), mobilizable or of prophage-like nature [5, 28].

Mobile Genetic Elements (MGEs) have likely played an important role in the genomic plasticity displayed by members of the *B. cereus* group. They consist of conjugative and mobilizable plasmids, such as pAW63 and pXO16 in *B. thuringiensis* [29, 30], Integrative and Conjugative Elements (ICEs) [31] and bacteriophages (phages). But they also include Transposable Elements (TEs) [32], such as Insertion Sequences (ISs, [33]), Class II transposons (Tns, [34]), Mobile Insertion Cassettes (MICs, [35]), Miniature Inverted repeat Transposable Elements (MITEs), Integrons [36], group II introns and *B. cereus* repeats (*BCRs*, [37]). ISs and Tn elements are often associated with virulence genes in *B. cereus s.l.* [38–40]. Each of these TEs presents a specific organization and transposition mechanism. ISs, MICs, Tns and MITEs all rely on the presence of an active transposase for mobility, whereas Integrons and ICEs use integrase and excisionase enzymes, as well as a recombination site.

ISs are prokaryotic transposable elements with a simple organization: two terminal inverted repeats (IR) flanking a transposase-coding gene, with certain exceptions such as those of the IS*200*/IS*605* family that display sub-terminal palindromic structures [41]. ISs are ubiquitous among prokaryotic genomes, with a differentiated distribution between chromosomes and plasmids [42, 43]. The complex class II transposable elements are represented in *B. cereus s.l.* by elements belonging to the Tn*3* family [34]. These elements, also flanked by terminal inverted repeats, carry a transposase and resolvase genes and occasionally one or more passenger genes.

The first *BCR* element was shown to exhibit mobile DNA features and to be restricted to the *B. cereus s.l.* group. Seventeen more elements were identified, characterized and divided into 3 groups: group A (*bcr1 - bcr3*), B (*bcr4 - bcr6*) and C (*bcr7 - bcr18*) [44]. The mobility of these elements is associated with a possible site-specific recombination-like mechanism, associated with the presence of one or more repetitions of a “TTTAT” motif.

As for group II introns, they are ubiquitous among bacterial genomes. Group I and II introns, discovered more than 20 years ago, are self-splicing catalytic RNAs, widespread in all bacterial genomes, including those of *B. cereus s.l.* They are characterized by complex secondary RNA structures, which play a key role in their mobility [45, 46]. The majority of group II introns encode IEPs (Intron Encoded Proteins), which insure their reverse transcription and aid in their movement within the genome [47, 48]. Group II introns are divided into three main families of RNA secondary structures: IIA, IIB and IIC [47].

In the present study, 102 complete and annotated *B. cereus s.l.* genomes were analysed for the prevalence and diversity of their TEs. This included the nine *B. cereus s.l.* species that displayed at least one complete genome available in the database. The analysed MGE types were IS elements, class II transposable elements belonging to the Tn*3* family, consensus *BCR* sequences and group II introns. A particular interest was also set on elements present on *B. thuringiensis* toxin-carrying plasmids.

## Results

### *B. cereus* sensu lato *genomes*

One hundred and two complete genome sequences belonging to *B. cereus s.l.* were retrieved from the NCBI (National Center for Biotechnology Information) genome database (Table 1). Of the forty-two complete genomes assigned in the NCBI database as *B. thuringiensis*, eight strains did not contain any delta-endotoxins and were marked as “cry- ”(crystal-). These strains included Bt407 and BMB171, two derivatives of wild-type crystalliferous strains, artificially cured by cultures at high temperatures [49, 50]. The six other strains included the debated pathogenic *B. thuringiensis* sv. *konkukian* strain 97–27, which should have been referred to as *B. cereus* [29]*.* As for *B. cereus s.s.,* four strains contained parts of *cry* or *cyt* (cytotoxin) coding genes. The accession numbers of the analysed genomes and the details of the *B. thuringiensis* cry- and *B. cereus* cry+ strains, grouped together as *B. thuringiensis*-like, are given in (Additional file 1: Table S1).

**Table 1:** Genomic details of the analyzed *B. cereus s.l.* species

	Number of genomes	Average chromosome size per strain (bp)	Average plasmid number per strain	Minimum-Maximum plasmid number per strain ^a^	Minimum-Maximum plasmid length per strain (bp) ^a^	Plasmid percentage of the genome (%)
*B. thuringiensis*	36	5,534,498	6.4	1–14	9261–761,374	11.2
*B. cereus* sensu stricto	42	5,312,889	2	1–7	2931–715,614	4.21
*B. thuringiensis*-like	8	5,417,908	1.8	1–6	14,888–591,112	7.56
*B. anthracis*	6	5,223,964	1.8	1–2	94,758–181,793	4.75
*B. mycoides*	4	5,327,723	5.5	3–8	4602–460,379	9.49
*B. weihenstephanensis*	2	5,435,562	2.0	NR	NR	5.31
*B. pseudomycoides*	1	NR	NR	NR	NR	0
*B. cytotoxicus*	1	NR	NR	NR	NR	0.17
*B. toyonensis*	1	NR	NR	NR	NR	1.69
*B. cereus* biovar *anthracis*	1	NR	NR	NR	NR	5.30

*NR* Not Relevant

^a^Some strains do not contain plasmids and were not taken into account

The most striking difference between species resides at the level of their plasmid content. This is particularly remarkable in the case of *B. thuringiensis* strains that harbour an average of 6.4 plasmids per strain and contain 11.2% of plasmidial DNA, as compared to the *B. cereus s.s.* strains with an average of two plasmids per strain, totalizing only 4.2% of plasmid DNA (Table 1).

### IS and Tn3 elements

**Fig. 1:**
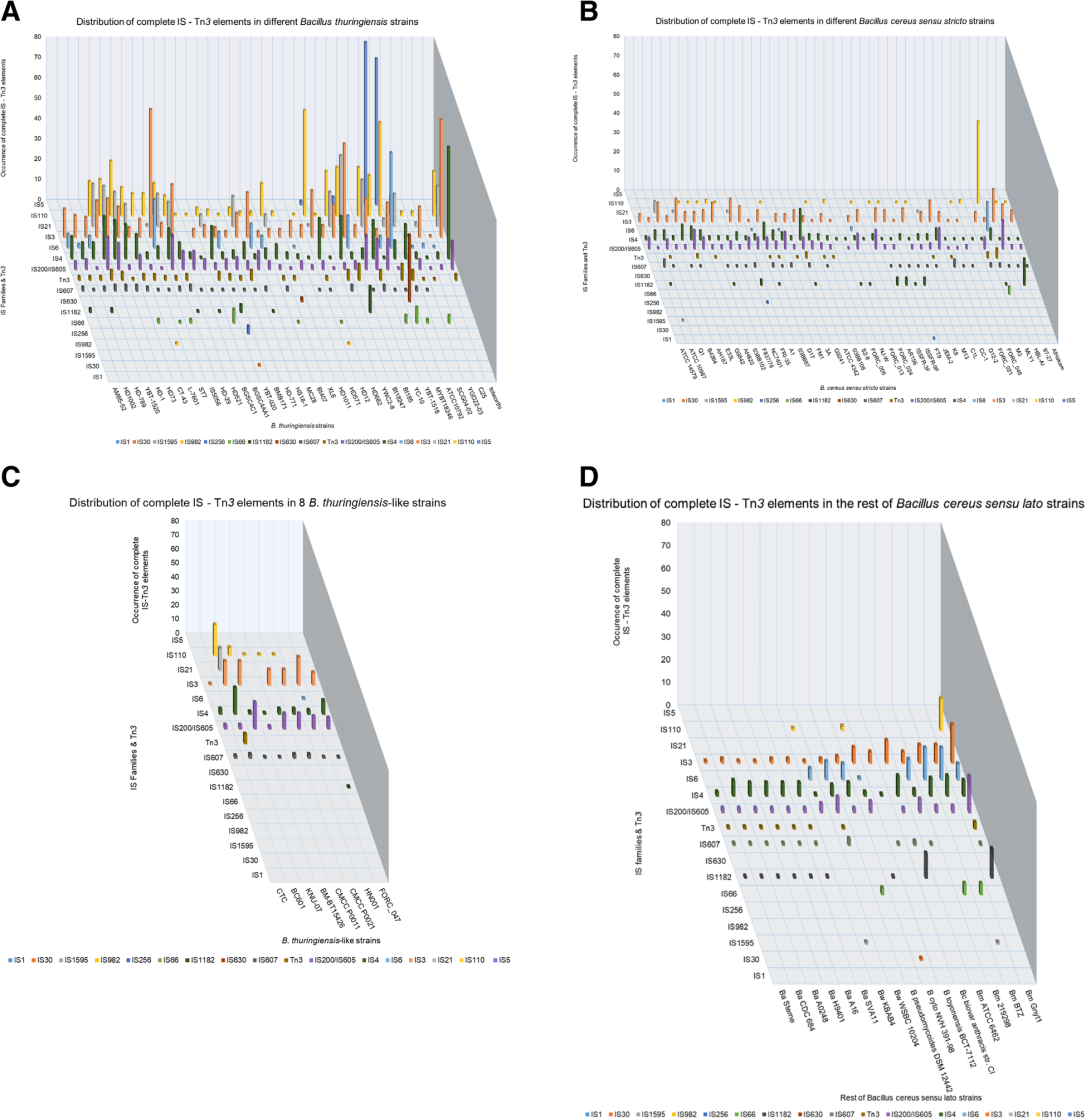
Distribution of complete IS family members and Tn*3* elements among the complete genomes of *B. cereus s.l*. The ISsaga web tool [51] was used for IS annotation. The number of complete ISs was detected in each strain's genome. Only families with at least one complete IS located on either the chromosome or plasmids are shown in this graphic representation. **a** IS distribution in 36 *B. thuringiensis* genomes, (**b**) 42 *B. cereus s.s.*, (**c**) eight *B. thuringiensis*-like, and (**d**) six *B. anthracis,* four *B. mycoides,* two *B. weihenstephanensis*, one *B. pseudomycoides,* one *B. cytotoxicus,* one *B. toyonensis* and one *B. cereus* biovar *anthracis*

As shown in Fig. 1 (a - d), sixteen ISs and Tn*3* families are represented by at least one copy in *B. cereus s.l.* Four noteworthy observations can be made on these data. The first observation concerns the copy number of ISs and Tn*3-*related elements, which varies between the nine *B. cereus s.l.* species (Fig. 1). Our data showed that the number of IS copies of a family also varies among strains of the same species, where *B. thuringiensis* strains tend to have higher copy numbers for certain families. This is for instance the case for the IS*3* family. The copy number of its elements varies between one and 63 copies per strain with an average of 14.3 elements in *B. thuringiensis*, vs. (versus) a variation between one and 17 and an average of 5.5 elements in *B. cereus s.s.* (Fig. 1a and b, respectively).

**Fig. 2:**
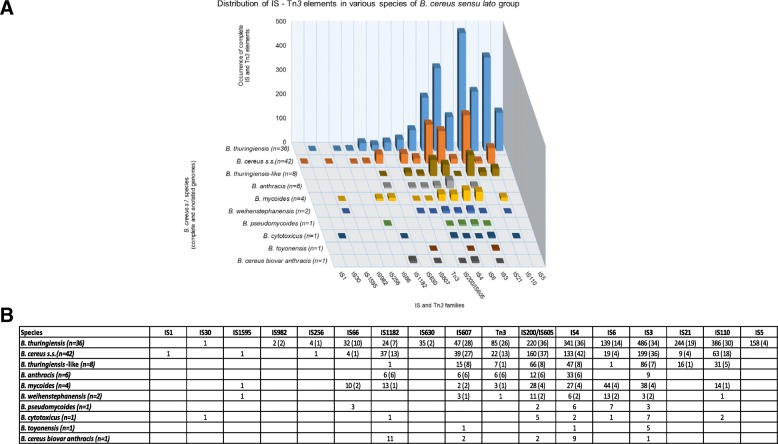
Distribution of the complete IS family members and Tn*3* elements among the species of *B. cereus s.l*. using the ISsaga web tool. The analysed number of complete genomes for each species is shown between parentheses. Only families with at least one complete IS located on either the chromosome or plasmids are shown in this (**a**) graphic representation of the total ISs and Tn*3* elements among the species of *B. cereus s.l*. and (**b**) the table detailing ISs and Tn*3* elements occurrence. Empty squares indicate the absence of the corresponding family and the number of positive strains for each family is shown between parentheses

The second observation is about the distribution of ISs and Tn*3*-like elements among the different species of the *B. cereus* group. Families such as IS*3*, IS*4* and IS*200*/IS*605* are ubiquitous among *B. cereus s.l.* while others, such as IS*1*, IS*30* and IS*982*, are rarely present (Figs. 1 and 2). Some families, despite their high copy number, are present in only a few strains. This is illustrated by the IS*5* family that has a total of 158 copies in *B. thuringiensis*, but unevenly distributed among only four strains (Fig. 2). Therefore, this IS family cannot be considered as a potential marker for *B. thuringiensis*. However, as for the widely distributed IS families, their higher copy number in *B. thuringiensis*, as a whole, distinguishes this species from the other *B. cereus* group members (Fig. 2).

**Fig. 3:**
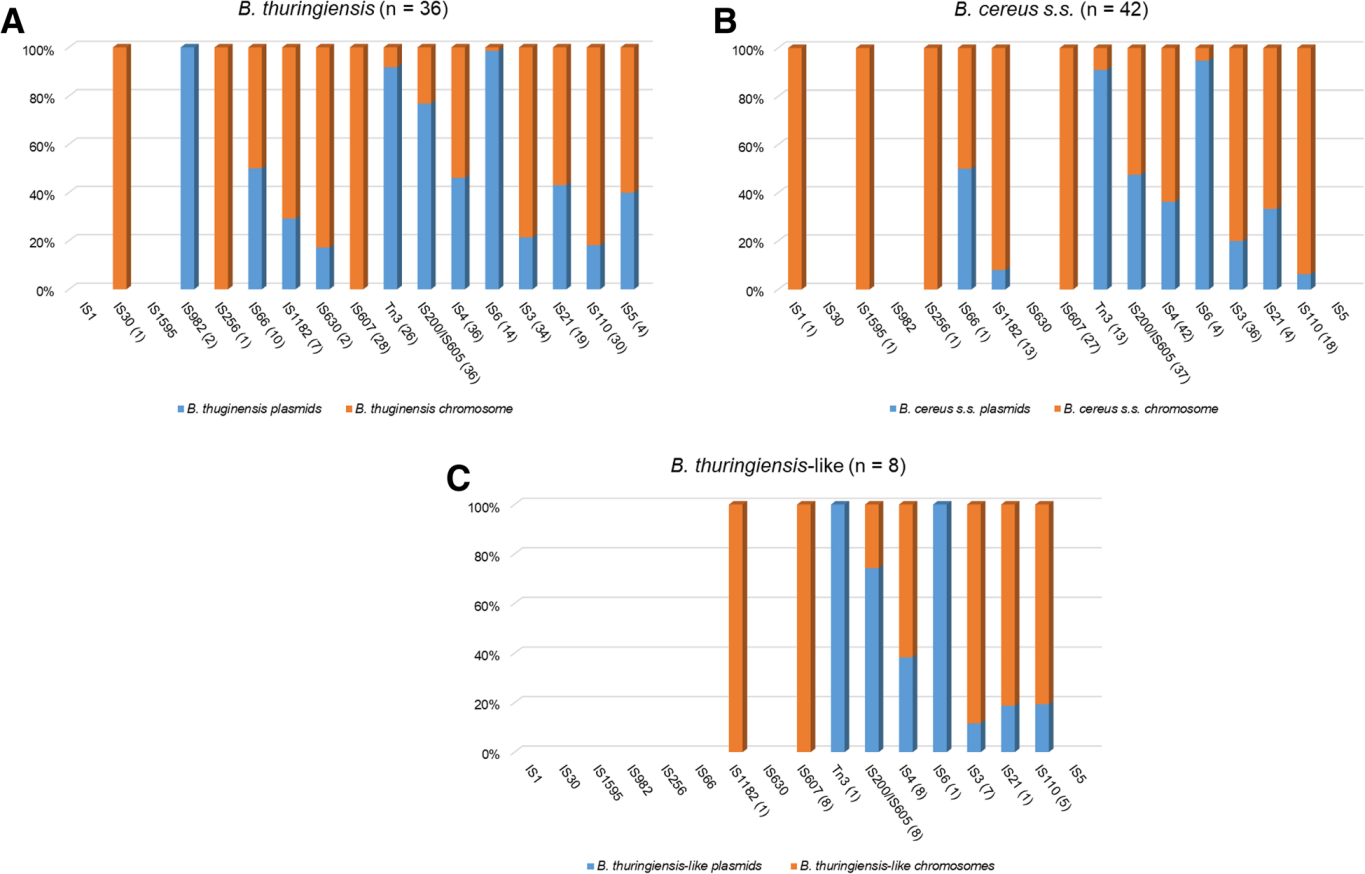
Distribution of ISs and Tn*3* family members between the chromosomes (red) and plasmids (blue) of *B. thuringiensis* (**a**)*, B. cereus s.s.* (**b**) and *B. thuringiensis*-like (**c**). The chromosomic and plasmidial distribution of each family member is shown in the graphs. The number of positive strains for each IS/Tn*3* family is indicated between parentheses

Thirdly, the distribution of these elements between the chromosomes and plasmids varies considerably according to the TE family. For instance, as shown in Fig. 3, IS*607* family is strictly chromosomic, in contrast to IS*6* family which is plasmid-borne at 100, 99 and 95% of the time for *B. thuringiensis*-like, *B. thuringiensis* and *B. cereus s.s.*, respectively. Members of the IS*4* family can be both chromosome- or plasmid-borne. As for Tn*3* family members, they are mostly found on plasmids (Fig. 3).

Finally, the average size of each family, the total number of ISs and Tn*3*-like elements present in each species provided us with a total ISs and Tn*3*-like elements size, used to calculate the percentage of TEs vis-à-vis of the total analysed genome, chromosome or plasmid size. As a whole, *B. thuringiensis* possesses the highest percentage (1.67%) of its genomes, which is related to ISs and Tn*3*-like elements, followed by *B. mycoides* (1.29%) and *B. cereus* biovar *anthracis* (0.93%). For *B. thuringiensis*, *B. cereus s.s.* and *B. anthracis*, the chromosomal vs. plasmidial IS/Tn*3*-like percentages varied greatly between the three species: while the chromosomal percentage is 1.10% in *B. thuringiensis,* 0.36% in *B. cereus s.s.* and 0.11% in *B. anthracis*, at the plasmidial level, *B. anthracis* has the highest percentage of 7.07%, followed by *B. thuringiensis* (6.18%) *B. thuringiensis*-like strains (4.50%) and finally *B. cereus* (3.73%).

### *B. cereus repeats* (BCRs)

Eighteen *BCR* repeated DNA elements, ranging from 100 to 400 bp, were also analysed in the complete genomes of *B. cereus s.l.* Their distribution among the 102 analysed *B. cereus s.l.* genomes is shown in (Additional file 1: Table S2). *Bcr1* is the most abundant element with a maximum of 130 integral copies in *B. mycoides* strains Gnyt1, a minimum of 11 copies in *B. anthracis* strains, and an average of 39 repeats per *B. cereus s.l.* strain. As for groups B and C, an average of two repeats per strain was found. *Bcr1, bcr4, bcr6, bcr10, bcr13* and *bcr16* have copies on either chromosomes or plasmids, whereas *bcr18* is only found on plasmids. Concerning the other eleven repeats, only chromosomal copies were found (data not shown).

An interesting observation was an apparent negative correlation between the *bcr1* and *bcr2* elements. A statistical analysis was conducted to find the presence or absence of a negative or positive correlation between *BCR* pairs for all the strains or by species. This analysis showed the existence of several significant correlations between pairs of *BCR* elements (*p* < 0.05; correlation coefficient is ≠ 0) (Additional file 1: Table S3). These correlation pairs, either positive or negative, vary in number among species. Twenty correlation pairs were found in *B. mycoides*, 25 in *B. cereus s.s.*, 14 in *B. thuringiensis* and 5 in *B. thuringiensis*-like (Additional file 1: Table S3-B). No significant correlation between *BCR* elements was found in *B. anthracis.*

Several correlation pairs were also common between species. Therefore the coefficients of these common pairs were compared via a linear regression model at a 95% confidence interval (Table 2) [52]. Between *B. mycoides* and *B. thuringiensis* and between *B. mycoides* and *B. cereus s.s.*, the common correlation pairs were significantly different. On the contrary, the differences between the common correlation pairs of *B. thuringiensis*, *B. thuringiensis*-like and *B. cereus s.s.* were not significant.

**Table 2:** Correlation coefficient of *BCRs* pairs between species

Compared species	Common correlating *BCRs* pairs	*p-value*	Status
*B. mycoides* vs. *B. cereus s.s*.	bcr1 - bcr2	0.0008	**S**
	bcr1 - bcr6	0.001	**S**
*B. mycoides* vs. *B. thuringiensis*	bcr1 - bcr2	0.0035	**S**
	bcr2 - bcr6	0.013	**S**
*B. thuringiensis* vs. *B. cereus s.s.*	bcr1 - bcr2	0.066	NS
	bcr1 - bcr5	0.913	NS
	bcr6-bcr13	0.673	NS
	bcr9-bcr11	0.592	NS
	bcr11-bcr14	0.861	NS
*B. thuringiensis*-like vs. *B. thuringiensis*	bcr1 - bcr5	0.169	NS
*B. thuringiensis*-like vs. *B. cereus s.s.*	bcr1 - bcr5	0.149	NS
	bcr1-bcr11	0.106	NS

*S* Significant difference, *NS* Non Significant difference

### Group II introns

The distribution of 30 known group II introns was analysed among the *B. cereus s.l.* genomes. The total number of group II introns varies from zero in *B. toyonensis* to 31 in strain HD29 of *B. thuringiensis* sv. *galleriae*. Although several group II introns were originally found in *B. cereus s.s.,* they were also observed in *B. thuringiensis* and *B. anthracis* genomes (Additional file 1: Table S4). However, there is a remarkable difference in the average copy number of group II introns per strain between the different species. For instance, *B. thuringiensis* has an average of nine group II introns per strain, whereas *B. cereus s.s.* displays a much lower average of three elements per strain, with 59 and 44% of the elements located on plasmids, for *B. thuringiensis* and *B. cereus s.s.*, respectively (Table 3). The presence of several copies of the same element (Additional file 1: Table S4) also suggests that it was once mobile. Another important observation is that most of group II introns located in *B. cereus s.l.* genomes belong to the RNA type IIB.

**Table 3:** Distribution of group II introns in *B. cereus s.l.* genomes

Species	Average intron number/strain	Percentage of introns located on plasmids (%)
*B. thuringiensis* (*n* = 36)	9	59
*B. cereus* sensu stricto (*n* = 42)	3	44
*B. thuringiensis*-like (*n* = 8)	3.4	45
*B. anthracis* (*n* = 6)	3	0
*B. mycoides* (*n* = 4)	2	25
*B. weihenstephanensis* (*n* = 2)	0	NA
*B. pseudomycoides* (n = 1)	2	100
*B. cytotoxicus* (n = 1)	1	100
*B. toyonensis* (n = 1)	0	NA
*B. cereus* biovar *anthracis* (*n* = 1)	3	0

*NA* Not Applicable

For *B. thuringiensis*, *B. cereus s.s.* and *B. thuringiensis*-like, group II introns are almost evenly distributed between chromosomes and plasmids, although with a slight bias (59%) towards plasmids for *B. thuringiensis* (Table 3). Of note, *B. anthracis* has only chromosomal group II introns.

### *A focus on B. thuringiensis toxin-carrying plasmids*

As mentioned above, virulence factors of the *B. cereus* group are often associated with TEs. In the case of *B. thuringiensis,* TEs found to be structurally associated with genes coding for delta-endotoxins include IS*231* [38, 53], IS*232* [54] and IS*240* [55]. Therefore, the distribution of ISs and Tn*3*-like elements, *BCRs* and group II introns was analysed on *B. thuringiensis* toxin-carrying plasmids. Note that *B. thuringiensis* strains HD682, HD1011, c25 and YGD22–03 were not considered, since they carry only a fragment of the *cry* or *cyt* (delta-endotoxin and cytolysin, respectively) chromosomal genes.

Even though they represent only 30.6% of the total *B. thuringiensis* plasmid repertoire, toxin-carrying plasmids hold 36.9, 49.4 and 44.4% of the total plasmidial ISs, Tn3-like elements, *bcr18* repeats and group II introns, respectively. Also, ISs and Tn*3*-like elements represent in size 7.46% of the toxin-carrying plasmids vs. 5.33% for the other plasmids.

## Discussion

*B. cereus s.l.* species are ecologically diverse but phylogenetically very close. Therefore, in this study, we aimed to explore the variety and distribution of MGEs in 102 complete *B. cereus s.l.* strains and analyse their possible contribution in the clustering of the group species. Three MGEs types were taken into consideration: ISs and Tn*3*-like elements, *Bacillus cereus* repeats and group II introns.

First and foremost, the genomes of *B. thuringiensis* and *B. cereus s.s.* were checked for the presence or absence of *cry*, *cyt* and *vip* (vegetative insecticidal protein) toxin genes. Some studies have indeed highlighted the importance of toxin-carrying plasmids in the phenotypical heterogeneity of the *B. cereus* group [23], while others have claimed that toxin-coding genes are not proof enough of a strain’s identity [21]. Based on several former phylogenetic studies, we chose to divide the analysed strains into *B. thuringiensis* (Cry+), *B. cereus s.s.* (Cry-) and *B. thuringiensis*-like (Additional file 1: Table S1).

Second, our data showed the distribution variability of ISs and Tn*3* elements among the different species, as well as among the different strains within the same species. For instance, *B. thuringiensis* presents an average of six plasmids per strain, with 6.18% of a plasmid being occupied by IS/Tn*3* elements, vs. an average of two plasmids per strain and 3.73% of IS/Tn*3* in *B. cereus s.s*. A strong positive correlation was found between TEs percentage per genome and the average number of plasmids per strain within a species, with a correlation coefficient of 0.91. This correlation and the above numbers indicate that ISs and Tn*3*-like elements play an important role in plasmid plasticity. This is of chief importance in the case of *B. thuringiensis* whose particular life style relies on the presence, diversity and combination of entomotoxin genes. Through their transposition mechanism and their ability to mobilize neighbouring DNA fragments, TEs together with conjugative plasmids are likely to participate in the necessary shuffling of the virulent genetic determinants of *B. thuringiensis*. In fact, these TEs are known to be associated with virulence genes [38, 39, 54–56], thus being linked to the variety of plasmid types. In fact, these TEs may transpose via mechanisms that lead to a co-integrate structure formation between conjugative and non-mobilizable plasmids, thus affecting plasmid mobility.

Also, although some IS/Tn*3* family patterns were noticeably different among the *B. cereus s.l.* species (e.g. IS*110* family, see Fig. 1), their differential distribution could not be used as marker to discriminate the different species within the *B. cereus* group. This is due to the fact that these elements are not ubiquitously present in all the strains of a particular species, and therefore cannot be used to distinguish among the different species (Fig. 2).

As for *BCRs*, the specific repeat elements found in the *B. cereus s.l.* group, 18 distinct repeat types were analysed for their copy number and genomic distribution. Apart from *bcr18*, they are mostly located on chromosomes. Their copy number differs between strains and *BCR* groups. These findings concur with those of previous studies regarding the general copy-number and distribution of the *BCR* repeats [44]. A noteworthy observation is the presence of significant correlations between different *BCR* pairs, which varied from one species to another. These correlations further confirm the tight phylogenetic association between *B. thuringiensis* and *B. cereus s.s.*, and the clear distinction between these species and *B. mycoides.* Consequently, based on the correlation pair analysis, the copy numbers of the distinct repeat types, as well as the mostly chromosomic location of these elements, the 102 *B. cereus s.l.* strains, could be divided into three groups: group 1 (*B. mycoides, B. weihenstephanensis* and *B. pseudomycoides*), group 2 (*B. thuringiensis, B. toyonensis, B. cereus s.s., B. cereus* biovar *anthracis* and *B. anthracis*) and group 3 which contains the divergent *B. cytotoxicus* strain.

The catalytic RNA group II introns showed no differential distribution among the *B. cereus s.l.* species on the level of intron-species specificity, but are present in a higher copy number in the genomes of *B. thuringiensis* strains. Some group II introns are equally distributed between plasmids and chromosomes, and others are specific to either the chromosome or plasmids. Our analysis also indicated that some elements are inserted in non-coding regions (e.g. B.c.I1 and B.my.I1) while others are inserted into functional CDS (Coding DNA Sequence) (data not shown). Two examples are B.th.I1 and B.th.I2 located on the *B. thuringiensis* sv. *kurstaki* conjugative plasmid pAW63 (DQ025752.1), within a putative hydrolase and a VirD4-like component, respectively. B.th.I1 encodes an endonuclease, while B.th.I2 is an CDS-less intron [57].

A principal component analysis (PCA) using the copy numbers of the various TEs, following a Chord transformation of the data [58], was unable to establish the species delineation (data not shown). This indicates that a phylogenetic analysis based only on TEs distribution is not enough on its own, and should to be combined with other approaches for more significant results.

Toxin-carrying plasmids are one of many plasmid types found in *B. thuringiensis* strains. These virulent plasmids are not only vital for *B. thuringiensis* insecticidal activity, but they are also important for other cellular functions such as sporulation, germination or horizontal gene transfer [5]. This is particularly true for the *B. thuringiensis* toxin-carrying plasmids that represent 30.6% of the plasmid pool. Interestingly, these plasmids carry a large portion of the total plasmidial IS-Tn*3*-like elements, *BCRs* and group II introns, with 7.46% (in size) of the toxin-carrying plasmids being ISs and Tn*3*-like elements vs. 5.33% for the other plasmids. As indicated above, this observation strongly suggests that these TEs elements have somehow contributed to the diversity of *B. thuringiensis* virulence, and hence its adaptation to the insect host spectrum. This is in line with previous observations linking ISs and Tn*3*-like elements to *cry* and *cyt* toxin genes and the presumed contribution of the formers in the mobility of the latters [38, 56, 59].

The variety and distribution of these mobile elements in other bacterial species is noteworthy. Concerning ISs and Tn*3*-like elements, the families described here can be found in several other species. An example is the well-known IS*4* family [60]. Members of this family are abundant in the *B. cereus s.l.* group (Fig. 2). In total, the latest data in the ISFinder database states that this family is found in 204 Gram-positive and Gram-negative bacterial species as well as in archaea. As indicated above, the *BCR* repeats are specific to the *B. cereus* group, and have not been found in any other species including those belonging to the *Bacillus* genera.

## Conclusion

This study underlines the great diversity of ISs and Tn*3*-like elements, *BCRs* and group II introns among strains and species of the *B. cereus s.l.* group. We took special care in analysing “clean” bacterial species, as in Cry+ *B. thuringiensis* and Cry- *B. cereus s.s.*, not relying solely on the database division. This study is therefore the first to analyse the distribution of more than one TE type in 102 complete *B. cereus s.l.* genomes. Analysis of these TEs is in agreement with previous phylogenetic studies that were unable to draw a clear line between *B. cereus s.s.* and *B. thuringiensis.* Finally, although mobile elements are not reliable on their own for establishing clear-cut phylogenetic clades, some of them, especially *BCR* elements, may serve as additional markers used alongside whole genome-based approaches.

## Methods

### Genomes

The genomes of *B. cereus s.l.* analysed in this study are completely sequenced, assembled and annotated and were downloaded from the NCBI genome database [68]. Since repeated elements may impair the complete assembly of a genome and are often located on the extremities of a contig or a scaffold, draft genomes with a contig or scaffold level assembly were not considered. In total, 42 *B. thuringiensis*, 44 *B. cereus s.s.*, 6 *B. anthracis*, 4 *B. mycoides,* 2 *B. weihenstephanensis* genomes and one genome of *B. cytotoxicus, B. pseudomycoides, B. toyonensis* and *B. cereus biovar anthracis* were analysed for their content in IS elements, class II transposable elements belonging to the Tn*3* family, consensus *BCR* sequences and group II introns (Table 1 and Additional file 1: Table S1). Since *B. anthracis* strains are highly clonal, only six were chosen randomly from the fifty complete genomes available in the NCBI genome database. Given the close phylogenetic relationship between *B. thuringiensis* and *B. cereus s.s.*, all their genomes were also inspected for the presence of delta-endotoxin coding genes. This was done by MegaBLAST search of toxins from 74 *cry*, three *cyt* and three *vip* gene families from the ad hoc database [61], further dividing the genome ensemble for each species into cry+ or cry- strains (e.g. *B. thuringiensis* vs. *B. thuringiensis* cry- and *B. cereus* vs. *B. cereus* cry+). Therefore *B. thuringiensis* cry- and *B. cereus* cry+ strains were grouped and assigned as *B. thuringiensis*-like.

### *Detection of ISs and Tn*3*-like elements*

Despite their small size and simple organization, ISs are rarely annotated correctly in sequenced genomes. A correct ISs identification requires both DNA and protein levels. Therefore, the ISFinder team has developed ISsaga (Insertion Sequence semi-automatic genome annotation) [51]. This online tool allows the annotation of complete or partial ISs as well as Tn*3* family elements. It first identifies the IS-associated CDSs and then runs a BLASTX analysis to confirm all the potential transposases. This analysis is followed by a cross-reference of the obtained nucleotide sequences with the ISFinder database [62].

For this study, ISsaga was used to annotate the complete genomes (chromosomes and plasmids) in “.Fasta” format and subsequently to conduct a manual verification of the results. This allowed us to extract the copy number of complete ISs and Tn*3* elements and to calculate their percentage in each genome.

### *Detection of* BCRs *and group II intron elements*

Analysis of the “*Bacillus cereus* repeats” (*BCR*) was done using nucleotide BLAST searches of the con*sensu*s *bcr1 - bcr18* [44] sequences against complete *B. cereus s.l.* genomes. Based on former studies [44, 63], and based on our observations, the algorithm parameters for MegaBLAST [64, 65] were set as follow: the word size - length of a seed that allows the BLAST engine to initiate an alignment - was set at 16; the opening and extension of a gap were both set at 2; expect range was set between 0 and 0.1. Hits that covered at least 50% of the sequence length, and had a minimum of 75% identity were considered as repeats. Correlation between the various *BCRs* repeats was calculated using JMP® (JMP®, version Pro 13. SAS institute Inc., NC. 1989–2007). The significance of the correlation between *BCRs* pairs for each species was then evaluated at a 95% confidence level. Species with only one or two complete genomes were excluded from this statistical analysis.

The same nucleotide MegaBLAST parameters were used to retrieve known group II introns from the bacterial group II intron database (http://webapps2.ucalgary.ca/~groupii/, [66] [67] in addition to *B.th.*I3i [48] which was absent from the mentioned database. This includes 27 elements with CDSs coding the IEP and three CDS-less elements.

## Additional file

Additional file 1: **Table S1A.** Accession numbers of the analyzed *B. cereus s.l.* replicons. *B. thuringiensis* cry- and *B. cereus s.s.* cry+ are placed under *B. thuringiensis-*like. **Table S1B.** Genomic details of eight *B. thuringiensis cry-* and four *B. cereus s.s. cry +* **Table S2.** Heat map of the total bcr number in the complete genomes of nine *B. cereus s.l.* species. The heat map is arranged by species and decreasing *bcr1* occurrence. **Table S3A.** Correlation pairs for the 102 analyzed *B. cereus s.l.* genomes. **Table S3B.** Correlation pairs for the 102 analyzed *B. cereus s.l.* genomes by species. **Table S4A.** Heat map and distribution of group II intron types in the complete genomes of 9 *B. cereus* sensu *lato* species. **Table S4B.** Heat map and distribution of group II introns in the complete genomes of 9 *B. cereus sensu lato* species. (DOCX 1276 kb)

## Abbreviations

*B.*: *Bacillus*
*BCR(s)*: *Bacillus cereus* repeat(s)
CDS(s): Coding DNA Sequence(s)
*cry*: crystal or delta-endotoxin gene
*cyt*: cytotoxin gene
GTEvaluator: Genome Target Evaluator
ICE(s): Integrative and Conjugative Element(s)
IEP(s): Intron Encoded Protein(s)
IR(s): Inverted Repeat(s)
IS(s): Insertion Sequence(s)
ISsaga: Insertion Sequence semi-automatic genome annotation
MGE(s): Mobile Genetic Element(s)
MIC(s): Mobile Insertion Cassette(s)
MITE(s): Miniature Inverted repeat Transposable Element(s)
MLST: Multi Locus Sequence Typing
NCBI: National Center for Biotechnology Information
PCA: Principal Component Analysis
*s.l.*: sensu *lato*
*s.s.*: sensu stricto
sv.: serovar
TE(s): Transposable Element(s)
Tn(s): class II transposons
*vip*: vegetative insecticidal protein gene
vs.: versus
