# Supplementary material for: Diversity of Bacillus cereus sensu lato mobilome
Source: BMC Genomics. 2019 May 29;20:436. doi: 10.1186/s12864-019-5764-4 (PMC6542083; doi:10.1186/s12864-019-5764-4)
Supplement: Supplementary file 1 — Table S1A. Accession numbers of the analyzed B. cereus s.l. replicons. B. thuringiensis cry- and B. cereus s.s. cry+ are placed under B. thuringiensis-like. Table S1B. Genomic details of eight B. thuringiensis cry- and four B. cereus s.s. cry + Table S2. Heat map of the total bcr number in the complete genomes of nine B. cereus s.l. species. The heat map is arranged by species and decreasing bcr1 occurrence. Table S3A. Correlation pairs for the 102 analyzed B. cereus s.l. genomes. Table S3B. Correlation pairs for the 102 analyzed B. cereus s.l. genomes by species. Table S4A. Heat map and distribution of group II intron types in the complete genomes of 9 B. cereus sensu lato species. Table S4B. Heat map and distribution of group II introns in the complete genomes of 9 B. cereus sensu lato species. (DOCX 1276 kb) [file 12864_2019_5764_MOESM1_ESM.docx]

Additional file 1: Table S1A: Accession numbers of the analyzed *B. cereus s.l.* replicons. *B. thuringiensis* cry- and *B. cereus s.s.* cry+ are placed under *B. thuringiensis-*like.

| ***B. thuringiensis* (n=36)** | | |
| --- | --- | --- |
| Strains | Molecule | Accession # |
| AM65-52 | Chromosome | CP013275.1 |
|  | pAM65-52-1 | CP013276.1 |
|  | pAM65-52-2 | CP013277.1 |
|  | pAM65-52-3 | CP013278.1 |
|  | pAM65-52-4 | CP013279.1 |
|  | pAM65-52-5 | CP013280.1 |
|  | pAM65-52-6 | CP013281.1 |
|  | pAM65-52-7 | CP013282.1 |
|  | pAM65-52-8 | CP013283.1 |
|  | pAM65-52-9 | CP013284.1 |
| HD1002 | Plasmid 7 | CP009344.1 |
|  | Plasmid 6 | CP009345.1 |
|  | Plasmid 5 | CP009346.1 |
|  | Plasmid 3 | CP009347.1 |
|  | Plasmid 2 | CP009348.1 |
|  | Plasmid 1 | CP009349.1 |
|  | Plasmid 4 | CP009350.1 |
|  | Chromosome | CP009351.1 |
| HD-789 | Chromosome | CP003763.1 |
|  | p01 | CP003764.1 |
|  | p02 | CP003765.1 |
|  | p03 | CP003766.1 |
|  | p04 | CP003767.1 |
|  | p05 | CP003768.1 |
|  | p06 | CP003769.1 |
| YBT-1520 | Chromosome | CP004858.1 |
|  | pBMB2062 | CP004859.1 |
|  | pBMB422 | CP004860.1 |
|  | pBMB293 | CP004861.1 |
|  | pBMB53 | CP004862.1 |
|  | pBMB11 | CP004863.1 |
|  | pBMB8513 | CP004864.1 |
|  | pBMB8240 | CP004865.1 |
|  | pBMB7921 | CP004866.1 |
|  | pBMB7635 | CP004867.1 |
|  | pBMB94 | CP004868.1 |
|  | pBMB67 | CP004869.1 |
| HD-1 | Chromosome | CP004870.1 |
|  | pBMB46 | CP004871.1 |
|  | pBMB64 | CP004872.1 |
|  | pBMB65 | CP004873.1 |
|  | pBMB74 | CP004874.1 |
|  | pBMB95 | CP004875.1 |
|  | pBMB299 | CP004876.1 |
|  | pBMB431 | CP004877.1 |
|  | pBMB2062 | CP004878.1 |
|  | pBMB7635 | CP004879.1 |
|  | pBMB8240 | CP004880.1 |
|  | pBMB8513 | CP004881.1 |
|  | pBMBLin15 | CP004882.1 |
|  | pBMB14 | CP004883.1 |
| HD73 | Chromosome | CP004069.1 |
|  | pAW63 | CP004072.1 |
|  | pHT11 | CP004073.1 |
|  | pHT7 | CP004076.1 |
|  | pHT73 | CP004070.1 |
|  | pHT77 | CP004071.1 |
|  | pHT8_1 | CP004074.1 |
|  | pHT8_2 | CP004075.1 |
| CT-43 | Chromosome | CP001907 |
|  | pCT127 | CP001908 |
|  | pCT14 | CP001909 |
|  | pCT281 | CP001910 |
|  | pCT51 | CP001911 |
|  | pCT6880 | CP001912 |
|  | pCT72 | CP001913 |
|  | pCT8252 | CP001914 |
|  | pCT83 | CP001915 |
|  | pCT8513 | CP001916 |
|  | pCT9547 | CP001917 |
| L-7601 | Chromosome | CP020002.1 |
|  | Plasmid 1 | CP020003.1 |
|  | Plasmid 2 | CP020004.1 |
|  | Plasmid 3 | CP020005.1 |
| ST7 | Chromosome | CP016194.1 |
|  | pST7-1 | CP016195.1 |
|  | pST7-2 | CP016196.1 |
|  | pST7-3 | CP016197.1 |
|  | pST7-4 | CP016198.1 |
| IS5056 | Chromosome | CP004123.1 |
|  | pIS56-6 | CP004124.1 |
|  | pIS56-8 | CP004125.1 |
|  | pIS56-9 | CP004126.1 |
|  | pIS56-11 | CP004127.1 |
|  | pIS56-15 | CP004128.1 |
|  | pIS56-16 | CP004129.1 |
|  | pIS56-39 | CP004130.1 |
|  | pIS56-63 | CP004131.1 |
|  | pIS56-68 | CP004132.1 |
|  | pIS56-85 | CP004133.1 |
|  | pIS56-107 | CP004134.1 |
|  | pIS56-233 | CP004135.1 |
|  | pIS56-285 | CP004136.1 |
|  | pIS56-328 | CP004137.1 |
| HD-29 | Chromosome | CP010089.1 |
|  | pBMB426 | CP010090.1 |
|  | pBMB267 | CP010091.1 |
|  | pBMB126 | CP010092.1 |
|  | pBMB71 | CP010093.1 |
|  | pBMB55 | CP010094.1 |
|  | pBMB47 | CP010095.1 |
|  | pBMBLin15 | CP010096.1 |
|  | pBMB12 | CP010097.1 |
|  | pBMB7 | CP010098.1 |
|  | pBMB8 | CP010099.1 |
| HD521 | Chromosome | CP010106.1 |
|  | pBTHD521-1 | CP010107.1 |
|  | pBTHD521-2 | CP010108.1 |
|  | pBTHD521-3 | CP010109.1 |
|  | pBTHD521-4 | CP010110.1 |
|  | pBTHD521-5 | CP010111.1 |
|  | pBTHD521-6 | CP010112.1 |
| BGSC4C1 | Chromosome | CP015176.1 |
|  | pBMB267 | CP015177.1 |
|  | pBMB4227 | CP015178.1 |
|  | pBMB57 | CP015179.1 |
|  | pBMB71 | CP015180.1 |
|  | pBMB9829 | CP015181.1 |
|  | pBMB8291 | CP015182.1 |
| BGSC4AA1 | Chromosome | CP010577 |
|  | pBMB232 | CP010578 |
|  | pBMB48 | CP010579 |
|  | pBMB51 | CP010580 |
|  | pBMB68 | CP010581 |
|  | pBMB76 | CP010582 |
|  | pBMB92 | CP010583 |
| YBT-020 | Chromosome | CP002508 |
|  | pBMB26 | CP002509 |
|  | pBMB28 | CP002510 |
| BMB171 | Chromosome | CP001903 |
|  | pBMB171 | CP001904 |
| HD-771 | Chromosome | CP003752 |
|  | p01 | CP003753 |
|  | p02 | CP003754 |
|  | p03 | CP003755 |
|  | p04 | CP003756 |
|  | p05 | CP003757 |
|  | p06 | CP003758 |
|  | p07 | CP003759 |
|  | p08 | CP003760 |
| HS18-1 | Chromosome | CP012099.1 |
|  | pHS18-1 | CP012100.1 |
|  | pHS18-2 | CP012101.1 |
|  | pHS18-3 | CP012102.1 |
|  | pHS18-4 | CP012103.1 |
|  | pHS18-5 | CP012104.1 |
|  | pHS18-6 | CP012105.1 |
|  | pHS18-7 | CP012106.1 |
|  | pHS18-8 | CP012107.1 |
|  | pHS18-9 | CP012108.1 |
| MC28 | Chromosome | CP003687.1 |
|  | pMC8 | CP003688.1 |
|  | pMC54 | CP003689.1 |
|  | pMC95 | CP003690.1 |
|  | pMC183 | CP003691.1 |
|  | pMC189 | CP003692.1 |
|  | pMC319 | CP003693.1 |
|  | pMC429 | CP003694.1 |
| Bt407 | Chromosome | CP003889.1 |
|  | BTB_502p | CP003890.1 |
|  | BTB_78p | CP003891.1 |
|  | BTB_15p | CP003892.1 |
|  | BTB_8p | CP003893.1 |
|  | BTB_7p | CP003894.1 |
|  | BTB_6p | CP003895.1 |
|  | BTB_5p | CP003896.1 |
|  | BTB_2p | CP003897.1 |
|  | BTB_9p | CP003898.1 |
| XL6 | Chromosome | CP013000.1 |
|  | Plasmid unnamed | CP013001.1 |
| HD1011 | Plasmid 3 | CP009332.1 |
|  | Plasmid 4 | CP009333.1 |
|  | Plasmid 2 | CP009334.1 |
|  | Chromosome | CP009335.1 |
|  | Plasmid 1 | CP009336.1 |
| HD571 | pBFQ | CP009599.1 |
|  | Chromosome | CP009600.1 |
| HD-12 | Chromosome | CP014847.1 |
|  | pHD120017 | CP014848.1 |
|  | pHD120038 | CP014849.1 |
|  | pHD120039 | CP014850.1 |
|  | pHD120112 | CP014851.1 |
|  | pHD120161 | CP014852.1 |
|  | pHD120345 | CP014853.1 |
| HD682 | pBGN_3 | CP009717.1 |
|  | pBGN_2 | CP009718.1 |
|  | pBGN_1 | CP009719.1 |
|  | Chromosome | CP009720.1 |
| YWC2-8 | Chromosome | CP013055.1 |
|  | pYWC2-8-1 | CP013056.1 |
|  | pYWC2-8-2 | CP013057.1 |
|  | pYWC2-8-3 | CP013058.1 |
|  | pYWC2-8-4 | CP013059.1 |
|  | pYWC2-8-5 | CP013060.1 |
|  | pYWC2-8-6 | CP013061.1 |
| Bt18247 | Chromosome | CP015250.1 |
|  | Plasmid p174778 | CP015251.1 |
|  | Plasmid p130548 | CP015252.1 |
|  | Plasmid p113275 | CP015253.1 |
|  | Plasmid p81952 | CP015254.1 |
|  | Plasmid p15092 | CP015255.1 |
|  | Plasmid p12509 | CP015256.1 |
| Bt185 | Chromosome | CP014282.1 |
|  | pBT1850636 | CP014283.1 |
|  | pBT1850294 | CP014284.1 |
|  | pBT1850055 | CP014285.1 |
|  | pBT1850054 | CP014286.1 |
|  | pBT1850042 | CP014287.1 |
|  | pBT1850012 | CP014288.1 |
|  | pBT1850046 | CP014289.1 |
|  | pBT1850007 | CP014290.1 |
| YC-10 | Chromosome | CP011349.1 |
|  | pYC1 | CP011350.1 |
|  | pYC3 | CP011351.1 |
|  | pYC4 | CP011352.1 |
|  | pYC5 | CP011353.1 |
|  | pYC6 | CP011354.1 |
|  | pYC10 | CP011355.1 |
|  | pYC11 | CP011356.1 |
|  | pYC20 | CP011357.1 |
|  | pYC2226 | CP011358.1 |
| YBT-1518 | Chromosome | CP005935 |
|  | pBMB0228 | CP002486 |
|  | pBMB0229 | CP005936 |
|  | pBMB0230 | CP005937 |
|  | pBMB0231 | CP005938 |
|  | pBMB0232 | CP005939 |
|  | pBMB0233 | CP005940 |
| MYBT18246 | Chromosome | CP015350.1 |
|  | p150790 | CP015351.1 |
|  | p142098 | CP015352.1 |
|  | p120510 | CP015353.1 |
|  | p120416 | CP015354.1 |
|  | p109822 | CP015355.1 |
|  | p101287 | CP015356.1 |
|  | p55166 | CP015357.1 |
|  | p46701 | CP015358.1 |
|  | p17175 | CP015359.1 |
|  | p14456 | CP015360.1 |
|  | p6330 | CP015361.1 |
| ATCC 10792 | Chromosome | CP021061.1 |
|  | Plasmid poh1 | CP021062.1 |
|  | Plasmid poh2 | CP021063.1 |
|  | Plasmid poh3 | CP021064.1 |
|  | Plasmid poh4 | CP021065.1 |
|  | Plasmid poh5 | CP021066.1 |
| SCG04-02 | Chromosome | CP017577.1 |
|  | Plasmid PSCG11 | CP017575.1 |
|  | Plasmid PSCG364 | CP017574.1 |
|  | Plasmid PSCG5 | CP017576.1 |
|  | Plasmid PSCG61 | CP017573.1 |
| YGD22-03 | Chromosome | CP019230.1 |
|  | Plasmid pYGD30 | CP019231.1 |
|  | Plasmid pYGD36 | CP019232.1 |
|  | Plasmid pYGD5 | CP019233.1 |
|  | Plasmid pYGD83 | CP019234.1 |
|  | Plasmid pYGD98 | CP019235.1 |
| Bt serovar Tolworthi  (Institut Pasteur standard strain) | Chromosome | AP014864.1 |
|  | pKK1 | AP014865.1 |
|  | pKK2 | AP014866.1 |
|  | pKK3 | AP014867.1 |
|  | pKK4 | AP014868.1 |
|  | pKK5 | AP014869.1 |
|  | pKK6 | AP014870.1 |
|  | pKK7 | AP014871.1 |
|  | pKK8 | AP014872.1 |
| c25 | chromosome | CP022345.1 |
|  | Plasmid unnamed1 | CP022346.1 |

| ***B. thuringiensis*-like**  **(n=8)** | | |
| --- | --- | --- |
| BM-BT15426 | Chromosome | CP020723.1 |
| CTC | Plasmid unnamed | CP013273.1 |
|  | Chromosome | CP013274.1 |
| Bc601 | Chromosome | CP015150.1 |
|  | pBTBC1 | CP015151.1 |
|  | pBTBC2 | CP015152.1 |
|  | pBTBC3 | CP015153.1 |
|  | pBTBC4 | CP015154.1 |
|  | pBTBC5 | CP015155.1 |
|  | pBTBC6 | CP015156.1 |
| KNU-07 | Chromosome | CP016588.1 |
|  | pBTKNU07-01 | CP016589.1 |
|  | pBTKNU07-02 | CP016590.1 |
| CMCC P0011 | Chromosome | CP011153.1 |
|  | Plasmid pRML05 | CP011154.1 |
| CMCC P0021 | Chromosome | CP011151.1 |
|  | pRML04 | CP011152.1 |
| FORC_047 | Chromosome | CP017060.1 |
|  | Plasmid pFORC47_1 | CP018741.1 |
|  | Plasmid pFORC47_2 | CP018742.1 |
| HN001 | Chromosome | CP011155.1 |
|  | pRML01 | CP011156.1 |
|  | pRML02 | CP011157.1 |

| ***B. cereus sensu stricto*** | | |
| --- | --- | --- |
| Strains | Molecule | Accession # |
| ATCC 14579 | Chromosome | AE016877.1 |
|  | pBClin15 | AE016878.2 |
| ATCC 10987 | Chromosome | AE017194.1 |
|  | pBc10987 | AE017195.1 |
| Q1 | Chromosome | CP000227.1 |
|  | pBc239 |  |
|  | pBc53 | CP000229.1 |
| B4264 | Chromosome | CP001176.1 |
| AH187 | Chromosome | CP001177.1 |
|  | pAH187_12 | CP001178.1 |
|  | pAH187_270 | CP001179.1 |
|  | pAH187_45 | CP001180.1 |
|  | pAH187_3 | CP001181.1 |
| E33L | Chromosome | CP009968.1 |
|  | pBCO_5 | CP009965.1 |
|  | pBCO_2 | CP009966.1 |
|  | pBCO_1 | CP009967.1 |
|  | pBCO_3 | CP009969.1 |
|  | pBCO_4 | CP009970.1 |
| G9842 | Chromosome | CP001186.1 |
|  | pG9842_209 | CP001187.1 |
|  | pG9842_140 | CP001188.1 |
| AH820 | Chromosome | CP001283.1 |
|  | pAH820_3 | CP001284.1 |
|  | pAH820_272 | CP001285.1 |
|  | pAH820_10 | CP001286.1 |
| 03BB102 | Plasmid unnamed | CP009317.1 |
|  | Chromosome | CP009318.1 |
| F837/76 | Chromosome | CP003187.1 |
|  | pF837_10 | CP003189.1 |
|  | pF837_55 | CP003188.1 |
| NC7401 | Chromosome | AP007209.1 |
|  | pNCcld | AP007210.1 |
|  | pNC1 | AP007211.1 |
|  | pNC2 | AP007212.1 |
|  | pNC3 | AP007213.1 |
|  | pNC4 | AP007214.1 |
| FRI-35 | Chromosome | CP003747.1 |
|  | p01 | CP003748.1 |
|  | p02 | CP003749.1 |
|  | p03 | CP003750.1 |
|  | p04 | CP003751.1 |
| A1 | Chromosome | CP015727.1 |
|  | pBCA2 | CP015728.1 |
|  | pBCA3 | CP015729.1 |
|  | pBCA1 | CP015730.1 |
| 03BB87 | pBCN | CP009939.1 |
|  | pBCX01 | CP009940.1 |
|  | Chromosome | CP009941.1 |
| D17 | Plasmid unnamed | CP009299.1 |
|  | Chromosome | CP009300.1 |
| FM1 | Plasmid unnamed | CP009368.1 |
|  | Chromosome | CP009369.1 |
| 3A | pBFC_1 | CP009593.1 |
|  | pBFC_2 | CP009594.1 |
|  | pBFC_3 | CP009595.1 |
|  | Chromosome | CP009596.1 |
| G9241 | pBFH_1 | CP009589.1 |
|  | Chromosome | CP009590.1 |
|  | pBC210 | CP009591.1 |
|  | pBCX01 | CP009592.1 |
| ATCC 4342 | pBGM | CP009627.1 |
|  | Chromosome | CP009628.1 |
| 03BB108 | pBFI_7 | CP009634.1 |
|  | pBFI_6 | CP009635.1 |
|  | pBFI_2 | CP009636.1 |
|  | pBFI_5 | CP009637.1 |
|  | pBFI_4 | CP009638.1 |
|  | pBFI_1 | CP009639.1 |
|  | pBFI_3 | CP009640.1 |
|  | Chromosome | CP009641.1 |
| S2-8 | pBFR_3 | CP009603.1 |
|  | pBFR_1 | CP009604.1 |
|  | Chromosome | CP009605.1 |
|  | pBFR_2 | CP009606.1 |
| FORC_005 | Chromosome | CP009686.1 |
| NJ-W | Chromosome | CP012483.1 |
|  | plasmid -35-36 | CP012484.1 |
|  | plasmid-32 | CP012485.1 |
|  | plasmid-37 | CP012486.1 |
| FORC_013 | Chromosome | CP011145.1 |
|  | pFORC13 | CP011146.1 |
| FORC_024 | Chromosome | CP012691.1 |
| AR156 | Chromosome | CP015589.1 |
|  | pAR10 | CP015590.1 |
|  | pAR41 | CP015591.1 |
|  | pAR460 | CP015592.1 |
| ISSFR-3F | Chromosome | CP018931.1 |
|  | Plasmid unnamed | CP018932.1 |
| ISSFR-9F | Chromosome | CP018933.1 |
|  | Plasmid unnamed1 | CP018934.1 |
| FT9 | Chromosome | CP008712.1 |
| JEM-2 | Chromosome | CP018935.1 |
|  | Plasmid unnamed1 | CP018936.1 |
| K8 | Chromosome | CP016595.1 |
|  | pBCM301 | CP016596.1 |
|  | pBCK802 | CP016597.1 |
| M13 | Chromosome | CP016360.1 |
|  | Plasmid pBCM1301 | CP016361.1 |
|  | Plasmid pBCM1302 | CP016362.1 |
|  | Plasmid pBCM1303 | CP016363.1 |
| C1L | Chromosome | CP022445.1 |
|  | Plasmid pC1L1 | CP022446.1 |
|  | Plasmid pC1L69 | CP022447.1 |
|  | Plasmid pC1L8 | CP022448.1 |
| CC-1 | Chromosome | CP023179.1 |
|  | Plasmid p1 | CP023180.1 |
|  | Plasmid p2 | CP023181.1 |
|  | Plasmid p3 | CP023182.1 |
|  | Plasmid p4 | CP023183.1 |
| D12_2 | Chromosome | CP016315.1 |
| FORC_021 | Chromosome | CP014486.1 |
|  | Plasmid unnamed | CP014487.1 |
| FORC_048 | Chromosome | CP017234.1 |
| M3 | Chromosome | CP016316.1 |
|  | Plasmid pBCM301 | CP016317.1 |
| MLY-1 | Chromosome MLY1.0 | CP024655.1 |
|  | Plasmid pMLY1.1 | CP024657.1 |
|  | Plasmid pMLY1.2 | CP024656.1 |
|  | Plasmid pMLY1.3 | CP024658.1 |
| Al Hakam | Chromosome | CP000485.1 |
|  | Plasmid pALH1 | CP000486.1 |
| 97-27 | Chromosome | CP010088.1 |
|  | Plasmid unnamed | CP010087.1 |

| ***B. anthracis*** | | |
| --- | --- | --- |
| Sterne | pXO1 | CP009540.1 |
|  | Chromosome | CP009541.1 |
| CDC 684 | pX02 | CP001214.1 |
|  | Chromosome | CP001215.1 |
|  | pX01 | CP001216.1 |
| A0248 | pXO2 | CP001597.1 |
|  | Chromosome | CP001598.1 |
|  | pXO1 | CP001599.1 |
| H9401 | Chromosome | CP002091.1 |
|  | BAP1 | CP002092.1 |
|  | BAP2 | CP002093.1 |
| A16 | Chromosome | CP001970.2 |
|  | pXO1 | CP001971.2 |
|  | pXO2 | CP001972.2 |
| SVA11 | Chromosome | CP006742.1 |
|  | pXO1 | CP006743.1 |
|  | pXO2 | CP006744.1 |

| ***B. mycoides*** | | |
| --- | --- | --- |
| *ATCC 6462* | pBMX_3 | CP009689.1 |
|  | pBMX_2 | CP009690.1 |
|  | pBMX_1 | CP009691.1 |
|  | Chromosome | CP009692.1 |
| *219298* | Plasmid unnamed_1 | CP007621.1 |
|  | Plasmid unnamed_2 | CP007622.1 |
|  | Plasmid unnamed_3 | CP007623.1 |
|  | Plasmid unnamed_4 | CP007624.1 |
|  | Plasmid unnamed_5 | CP007625.1 |
|  | Chromosome | CP007626.1 |
| *BTZ* | Chromosome | CP009651.1 |
|  | pBTZ_1 | CP009650.1 |
|  | pBTZ_2 | CP009649.1 |
|  | pBTZ_3 | CP009648.1 |
|  | pBTZ_4 | CP009647.1 |
|  | pBTZ_5 | CP009645.1 |
|  | pBTZ_6 | CP009646.1 |
| *Gnyt1* | Chromosome | CP020743.1 |
|  | Plasmid unnamed1 | CP020744.1 |
|  | Plasmid unnamed2 | CP020745.1 |
|  | Plasmid unnamed3 | CP020746.1 |
|  | Plasmid unnamed4 | CP020747.1 |
|  | Plasmid unnamed5 | CP020748.1 |
|  | Plasmid unnamed6 | CP020749.1 |
|  | Plasmid unnamed7 | CP020750.1 |
|  | Plasmid unnamed8 | CP020751.1 |

| ***Bacillus pseudomycoides*** DSM 12442 | Chromosome | CM000745.1 |
| --- | --- | --- |

| ***B. weihenstephanensis*** | | |
| --- | --- | --- |
| *KBAB4* | Chromosome | CP000903.1 |
|  | pBWB401 | CP000904.1 |
|  | pBWB402 | CP000905.1 |
|  | pBWB403 | CP000906.1 |
|  | pBWB404 | CP000907.1 |
| *WSBC 10204* | Chromosome | CP009746.1 |

| ***B. cytotoxicus*** NVH 391-98 | Chromosome | CP000764.1 |
| --- | --- | --- |
|  | Plasmid pBC9801 | CP000765.1 |

| ***B. toyonensis*** BCT-7112 | Chromosome | CP006863.1 |
| --- | --- | --- |
|  | pBCT77 | CP006864.1 |
|  | pBCT8 | CP006865.1 |

| ***B. cereus biovar anthracis str.*** CI | Chromosome | CP001746.1 |
| --- | --- | --- |
|  | pCI-XO1 | CP001747.1 |
|  | pCI-XO2 | CP001748.1 |
|  | pBAslCI14 | CP001749.1 |

Additional file 1: Table S1B: Genomic details of eight *B. thuringiensis cry-* and four *B. cereus s.s. cry+*

| Current Species | Strain | Replicons | Accession # | Sequencing Technique | Sequencing Lab | Notes | Conclusions |
| --- | --- | --- | --- | --- | --- | --- | --- |
| *B. thuringiensis* | Al Hakam | Chromosome | CP000485.1 | plasmid and cosmid DNA libraries | Los Alamos National Laboratory | pALH1 is a circular phage | Not *B. thuringiensis* |
|  |  | Plasmid pALH1 | CP000486.1 |  |  |  |  |
|  | 97-27 | Chromosome | CP010088.1 | Illumina; 454 | Los Alamos National Laboratory |  | Not *B. thuringiensis* |
|  |  | Plasmid unnamed | CP010087.1 |  |  |  |  |
|  | CTC | Plasmid unnamed | CP013273.1 | Illumina; Sanger | State Key Laboratory of Agricultural - Microbiology, Huazhong Agricultural University - China | No toxin coding genes but genomic analysis confirm it's *B. thuringiensis:* identified as serotype H2, serovar. *finitimus* with a high production of an S-layer protein which is part of a parasporal inclusion (Dong *et al.* 2016) | *B. thuringiensis*-like |
|  |  | Chromosome | CP013274.1 |  |  |  |  |
|  | Bc601 | Chromosome | CP015150.1 | Illumina | Key Laboratory of Systems Bioengineering - Tianjin University - China | No toxin coding genes but genomic analysis confirm it's *B. thuringiensis*: close to *B. thuringiensis* sv. *kurstaki* HD73. (Jia *et al.* 2016) | *B. thuringiensis*-like |
|  |  | pBTBC1 | CP015151.1 |  |  |  |  |
|  |  | pBTBC2 | CP015152.1 |  |  |  |  |
|  |  | pBTBC3 | CP015153.1 |  |  |  |  |
|  |  | pBTBC4 | CP015154.1 |  |  |  |  |
|  |  | pBTBC5 | CP015155.1 |  |  |  |  |
|  |  | pBTBC6 | CP015156.1 |  |  |  |  |
|  | KNU-07 | Chromosome | CP016588.1 | PacBio | School of Applied Biosciences - Kyungpook National University - South Korea | No toxin coding genes but genomic analysis confirm it's *B. thuringiensis*: ANI value for the genome comparaison and 16S rRNA gene. | *B. thuringiensis*-like |
|  |  | pBTKNU07-01 | CP016589.1 |  |  |  |  |
|  |  | pBTKNU07-02 | CP016590.1 |  |  |  |  |
|  | BM-BT15426 | Chromosome | CP020723.1 | PacBio | South China University of Technology | Only chromosome sequence Identified as *B. thuringiensis* after WGS by 16S comparaison and whole genome alignment | *B. thuringiensis*-like |
|  | Bt407 | Chromosome | CP003889.1 | Sanger dideoxy sequencing; 454 | Georg-August-University Goettingen, Genomic and Applied Microbiology; Goettingen Genomics Laboratory - Germany | Acrystalliferous derivative of a lepidopteran active strain, obtained through culturing at a high temperature. | *B. thuringiensis* |
|  |  | BTB_502p | CP003890.1 |  |  |  |  |
|  |  | BTB_78p | CP003891.1 |  |  |  |  |
|  |  | BTB_15p | CP003892.1 |  |  |  |  |
|  |  | BTB_8p | CP003893.1 |  |  |  |  |
|  |  | BTB_7p | CP003894.1 |  |  |  |  |
|  |  | BTB_6p | CP003895.1 |  |  |  |  |
|  |  | BTB_5p | CP003896.1 |  |  |  |  |
|  |  | BTB_2p | CP003897.1 |  |  |  |  |
|  |  | BTB_9p | CP003898.1 |  |  |  |  |
|  | BMB171 | Chromosome | CP001903 | Sanger dideoxy sequencing; 454; Illumina | State Key Laboratory of Agricultural - Microbiology, Huazhong Agricultural University - China | Acrystalliferous mutant obtained from wild-type crystalliferous strain YBT-146 through culturing at a high temp and adding 0,05% SDS. | *B. thuringiensis* |
|  |  | pBMB171 | CP001904 |  |  |  |  |
| *B. cereus sensu stricto* | CMCC P0011 | Chromosome | CP011153.1 | PacBio | National Institute for Food and Drug Control - China | In both cases: 99% Q & 33 % ID with Cry3 --> annotated in the plasmids as hypothetical protein | *B. thuringiensis*-like |
|  |  | Plasmid pRML05 | CP011154.1 |  |  |  |  |
|  | CMCC P0021 | Chromosome | CP011151.1 | PacBio | National Institute for Food and Drug Control - China |  |  |
|  |  | pRML04 | CP011152.1 |  |  |  |  |
|  | HN001 | Chromosome | CP011155.1 | PacBio | National Institute for Food and Drug Control - China | On a plasmid; 69 % Q & 23 % ID with Cyt1 --> In the annotation: Part of a hypothetical protein (start of the alignement is *in phase* with the start of the hypo prot) | *B. thuringiensis*-like |
|  |  | pRML01 | CP011156.1 |  |  |  |  |
|  |  | pRML02 | CP011157.1 |  |  |  |  |
|  | FORC_047 | Chromosome | CP017060.1 | PacBio | Food Science and Biotechnology - south korea | On a plasmid; 80 % Q & 24 % ID with Cry35 --> in the annotation: Part of a DNA topoisomerase. | *B. thuringiensis*-like |
|  |  | Plasmid pFORC47_1 | CP018741.1 |  |  |  |  |
|  |  | Plasmid pFORC47_2 | CP018742.1 |  |  |  |  |


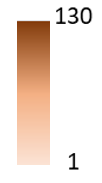
 Additional file 1: Table S2 Heat map of the total bcr number in the complete genomes of nine *B. cereus s.l.* species. The heat map is arranged by species and
decreasing bcr1 occurrence.

|  |  | **bcr1** | **bcr2** | **bcr3** | **bcr4** | **bcr5** | **bcr6** | **bcr7** | **bcr8** | **bcr9** | **bcr10** | **bcr11** | **bcr12** | **bcr13** | **bcr14** | **bcr15** | **bcr16** | **bcr17** | **bcr18** |
| --- | --- | --- | --- | --- | --- | --- | --- | --- | --- | --- | --- | --- | --- | --- | --- | --- | --- | --- | --- |
| *B. mycoides* | Gnyt1 | 130 | 7 | 29 | 2 | 2 | 2 |  | 1 | 2 | 2 | 1 | 2 | 4 | 3 | 2 | 6 | 2 | 2 |
|  | ATCC6462 | 104 | 2 | 16 | 3 | 1 |  |  | 1 | 2 | 2 | 1 | 2 | 12 | 3 | 2 | 1 | 2 |  |
|  | 219298 | 101 |  | 19 | 4 | 2 |  |  | 1 | 2 | 2 | 1 | 3 | 31 | 2 | 3 | 1 | 1 |  |
|  | BTZ | 101 |  | 22 | 3 | 2 |  |  | 1 | 2 | 2 | 1 | 3 | 32 | 2 | 3 | 1 | 1 |  |
| *B. weihenstephanensis* | WSBC 10204 | 119 | 4 | 29 | 2 | 1 | 2 |  | 1 | 2 | 2 | 1 | 2 | 2 | 3 | 2 | 3 | 2 |  |
|  | KBAB4 | 107 | 6 | 17 | 2 | 4 |  |  | 1 | 2 | 2 | 1 | 2 | 3 | 3 | 2 | 2 | 2 |  |
| *B. pseudomycoides* | DSM 12442 | 101 |  | 19 | 2 | 1 |  |  | 1 | 2 | 2 | 1 | 3 | 26 | 2 | 3 |  | 1 |  |
| *B. thuringiensis* | MC28 | 94 | 77 | 5 | 1 | 3 | 1 |  | 1 | 2 | 2 | 1 | 3 | 2 | 2 | 2 | 7 | 1 |  |
|  | BGSC 4AA1 | 93 | 23 | 25 | 2 | 3 | 3 |  | 1 | 3 | 3 | 2 | 1 | 2 | 2 | 2 | 2 | 2 | 7 |
|  | HD12 | 93 | 22 | 24 | 2 | 3 | 2 |  | 1 | 2 | 2 | 1 | 2 | 2 | 2 | 2 | 2 | 2 | 4 |
|  | HS18-1 | 90 | 39 | 12 | 2 |  |  |  | 1 | 2 | 2 | 1 | 1 | 2 | 3 | 2 | 1 | 2 |  |
|  | BGSC 4C1 | 87 | 27 | 14 | 3 | 2 | 2 |  | 1 | 2 | 2 | 1 | 2 | 2 | 3 | 2 | 2 | 2 | 3 |
|  | AM65-52 | 83 | 25 | 21 | 5 | 2 | 2 |  | 1 | 2 | 2 | 1 | 2 | 2 | 3 | 2 | 2 | 2 |  |
|  | HD1002 | 83 | 24 | 22 | 6 | 2 | 2 |  | 1 | 2 | 2 | 1 | 2 | 2 | 3 | 2 | 2 | 2 |  |
|  | HD-789 | 82 | 25 | 20 |  | 2 | 2 |  | 1 | 2 | 2 | 1 | 1 | 2 | 3 | 2 | 2 | 2 |  |
|  | L-7601 | 82 | 26 | 17 | 2 | 2 | 2 |  | 1 | 2 | 2 | 1 | 3 | 2 | 3 | 2 | 2 | 2 | 2 |
|  | HD-771 | 80 | 22 | 19 | 2 | 3 | 2 |  | 1 | 2 | 2 | 1 | 2 | 2 | 3 | 2 | 3 | 1 | 8 |
|  | XL6 | 64 | 36 | 13 |  | 2 | 1 |  | 1 | 2 | 2 | 1 | 2 | 2 |  | 2 | 2 | 2 |  |
|  | Bt185 | 61 | 46 | 18 | 2 | 1 | 2 |  | 1 | 2 | 2 | 1 | 3 | 3 | 3 | 2 | 3 | 2 | 8 |
|  | YBT-020 | 58 | 58 | 20 | 3 | 3 |  |  | 1 | 2 | 2 | 1 | 2 | 1 | 3 | 2 | 2 | 2 |  |
|  | HD-29 | 57 | 55 | 19 | 2 | 2 |  |  | 1 | 2 | 3 | 1 | 2 | 1 | 3 | 2 | 2 | 4 | 8 |
|  | tolworthi | 55 | 39 | 24 | 2 | 3 | 2 |  | 1 | 2 | 2 | 1 | 2 | 3 | 3 | 2 | 3 | 2 | 9 |
|  | YBT-1520 | 54 | 56 | 19 | 2 | 2 |  |  | 1 | 2 | 2 | 1 | 2 | 1 | 3 | 2 | 3 | 2 | 7 |
|  | HD-1 | 54 | 52 | 17 | 2 | 3 |  |  | 1 | 2 | 2 | 1 | 2 | 1 | 4 | 2 | 3 | 2 | 8 |
|  | HD73 | 54 | 56 | 19 | 5 | 2 |  |  | 1 | 2 | 2 | 1 | 2 | 1 | 3 | 2 | 2 | 2 |  |
|  | YWC2-8 | 54 | 56 | 19 | 2 | 2 |  |  | 1 | 2 | 2 | 1 | 2 | 1 | 3 | 2 | 2 | 2 | 3 |
|  | YC-10 | 54 | 56 | 19 | 2 | 2 |  |  | 1 | 2 | 2 | 1 | 2 | 1 | 3 | 2 | 3 | 2 | 9 |
|  | BMB171 | 52 | 34 | 19 |  | 5 | 1 |  | 1 | 2 | 3 | 1 | 1 | 4 | 3 | 2 | 2 | 2 |  |
|  | ST7 | 51 | 55 | 16 | 2 | 4 |  |  | 1 | 2 | 2 | 1 | 2 | 2 | 3 | 2 | 2 | 2 |  |
|  | HD521 | 50 | 53 | 20 | 2 | 4 |  |  | 1 | 2 | 2 | 1 | 2 | 2 | 3 | 2 | 2 | 2 |  |
|  | SCG04-02 | 50 | 41 | 26 |  | 6 |  |  | 2 | 2 | 2 | 1 | 2 | 3 | 3 | 2 | 2 | 2 |  |
|  | Bt c25 | 50 | 63 | 32 | 2 | 1 |  |  | 1 | 2 | 2 | 1 | 2 | 2 | 3 | 2 | 5 | 2 | 4 |
|  | IS5056 | 49 | 41 | 8 |  | 4 | 1 |  | 1 | 2 | 2 | 1 | 1 | 2 | 3 | 2 | 2 | 2 |  |
|  | Bt407 | 49 | 41 | 8 | 2 | 4 | 1 |  | 1 | 2 | 2 | 1 | 1 | 2 | 3 | 2 | 2 | 2 |  |
|  | ATCC10792 | 49 | 41 | 8 | 2 | 4 | 1 |  | 1 | 2 | 2 | 1 | 1 | 2 | 3 | 2 | 2 | 2 | 5 |
|  | YGD22-03 | 49 | 62 | 35 | 2 | 1 |  |  | 1 | 2 | 2 | 1 | 2 | 2 | 3 | 2 | 3 | 2 |  |
|  | CT-43 | 48 | 41 | 8 |  | 4 | 1 |  | 1 | 2 | 2 | 1 | 1 | 2 | 3 | 2 | 2 | 2 |  |
|  | YBT-1518 | 48 | 29 | 7 | 2 | 2 | 1 |  | 1 | 2 | 2 | 1 | 2 | 2 | 3 | 2 | 2 | 2 |  |
|  | Bt18247 | 46 | 32 | 9 | 2 | 2 | 1 |  | 1 | 2 | 2 | 1 | 2 | 2 | 2 | 2 | 2 | 2 |  |
|  | MYBT18246 | 44 | 31 | 8 | 2 |  | 2 |  | 1 | 2 | 2 | 1 | 1 | 2 | 3 | 2 | 2 | 2 |  |
|  | HD571 | 36 | 72 | 11 | 3 | 7 | 2 |  | 1 | 2 | 2 | 1 | 2 | 2 | 2 | 2 | 1 | 2 |  |
|  | HD1011 | 15 | 69 | 18 | 2 | 4 | 2 |  | 1 | 2 | 2 | 2 | 2 | 2 | 2 | 2 | 2 | 2 |  |
|  | HD682 | 15 | 65 | 14 | 2 | 5 | 1 |  | 1 | 2 | 2 | 2 | 2 | 2 | 2 | 2 | 2 | 2 |  |
| *B. toyonensis* | BCT-7112 | 96 | 38 | 8 | 2 | 1 | 1 |  | 1 | 2 | 2 | 1 | 2 | 1 | 3 | 2 | 1 | 1 |  |
| *B. cereus sensu stricto* | G9842 | 85 | 26 | 19 | 2 | 3 | 2 |  | 1 | 2 | 2 | 1 | 2 | 2 | 3 | 2 | 2 | 2 |  |
|  | M3 | 84 | 53 | 9 | 2 | 5 |  |  | 1 | 2 | 2 | 1 | 2 |  | 3 | 2 | 2 | 1 | 3 |
|  | ATCC 10987 | 77 | 46 | 11 | 3 |  |  |  | 1 | 2 | 3 | 1 | 1 | 2 | 3 | 2 | 1 | 2 | 4 |
|  | FRI-35 | 75 | 50 | 11 | 2 |  |  |  | 1 | 2 | 2 | 1 | 2 | 1 | 3 | 2 | 2 | 2 | 3 |
|  | AR156 | 71 | 40 | 12 | 2 | 2 |  |  | 1 | 2 | 2 | 1 | 2 | 2 | 3 | 2 | 2 | 2 |  |
|  | ATCC 4342 | 65 | 38 | 17 | 2 | 2 |  |  | 1 | 2 | 2 | 1 | 2 | 3 | 3 | 2 | 2 | 1 |  |
|  | C1L | 64 | 37 | 11 | 2 | 1 | 3 |  | 1 | 2 | 2 | 1 | 2 | 3 | 3 | 2 | 3 | 2 | 6 |
|  | D12_2 | 61 | 45 | 15 | 2 | 4 | 1 |  | 1 | 2 | 2 | 1 | 2 | 2 | 3 | 2 | 3 | 2 |  |
|  | ATCC 14579 | 60 | 32 | 19 | 2 | 2 | 1 |  | 1 | 2 | 2 | 1 | 1 | 3 | 3 | 2 | 2 | 2 |  |
|  | FM1 | 60 | 31 | 22 |  |  |  |  | 1 | 2 | 2 | 1 | 2 | 2 | 3 | 2 | 1 | 2 |  |
|  | 03BB87 | 58 | 31 | 21 | 2 |  |  |  | 1 | 2 | 2 | 1 | 2 | 1 | 3 | 2 | 1 | 2 | 3 |
|  | G9241 | 58 | 32 | 24 | 3 |  |  |  | 1 | 2 | 2 | 1 | 2 | 1 | 3 | 2 | 1 | 2 | 3 |
|  | HBL-AI | 57 | 40 | 19 | 2 | 7 | 4 |  | 1 | 2 | 2 | 1 | 2 | 4 | 3 | 2 | 3 | 2 | 5 |
|  | FORC_024 | 55 | 39 | 16 | 2 | 4 | 1 |  | 1 | 2 | 2 | 1 | 3 | 3 | 3 | 2 | 2 | 2 |  |
|  | FORC021 | 55 | 39 | 21 | 2 | 6 | 1 |  | 1 | 2 | 2 | 1 | 2 | 3 | 3 | 2 | 2 | 2 |  |
|  | FORC_048 | 55 | 46 | 17 | 2 | 4 |  |  | 1 | 2 | 2 | 1 | 2 | 2 | 3 | 2 | 3 | 2 |  |
|  | B4264 | 54 | 45 | 18 | 1 | 2 |  |  | 1 | 2 | 2 | 1 | 2 | 2 | 3 | 2 | 3 | 2 |  |
|  | A1 | 54 | 54 | 21 | 2 | 1 |  |  | 1 | 2 | 2 | 1 | 2 | 2 | 3 | 2 | 2 | 2 |  |
|  | FORC_005 | 53 | 40 | 16 | 2 | 4 | 1 |  | 1 | 2 | 2 | 1 | 2 | 2 | 3 | 2 | 3 | 2 |  |
|  | FORC_013 | 53 | 37 | 20 | 2 | 5 | 1 |  | 1 | 2 | 2 | 1 | 2 | 2 | 3 | 2 | 2 | 2 |  |
|  | FT9 | 52 | 20 | 7 | 2 |  |  |  | 1 | 1 | 2 |  | 1 | 1 | 2 | 2 | 1 | 2 |  |
|  | NJ_W | 51 | 40 | 10 | 10 | 2 | 1 |  | 1 | 2 | 2 | 1 | 2 | 2 | 3 | 2 | 2 | 2 |  |
|  | M13 | 51 | 48 | 18 | 2 | 4 | 1 |  | 1 | 2 | 2 | 1 | 2 | 3 | 3 | 2 | 2 | 2 |  |
|  | Q1 | 50 | 55 | 16 | 1 |  |  |  | 1 | 2 | 2 | 1 | 2 | 1 | 3 | 2 | 2 | 2 | 2 |
|  | K8 | 50 | 52 | 25 | 2 | 4 |  |  | 1 | 2 | 2 | 1 | 2 | 2 | 3 | 2 | 2 | 2 |  |
|  | AH187 | 48 | 46 | 17 | 1 | 2 |  |  | 1 | 2 | 2 | 1 | 2 | 1 | 3 | 2 | 2 | 1 | 4 |
|  | NC7401 | 48 | 66 | 18 | 2 |  |  |  | 1 | 2 | 2 | 1 | 2 | 2 | 3 | 2 | 2 | 1 | 4 |
|  | MLY1 | 45 | 40 | 14 | 2 | 1 | 1 |  | 1 | 2 | 2 | 1 | 2 | 3 | 3 | 2 | 3 | 2 |  |
|  | Al Hakam | 36 | 72 | 30 | 3 | 7 | 2 |  | 1 | 2 | 2 | 1 | 2 | 2 | 2 | 2 | 1 | 2 |  |
|  | 03BB108 | 36 | 79 | 14 | 3 | 7 | 2 | 1 | 1 | 2 | 3 | 1 | 2 | 1 | 2 | 2 | 1 | 2 | 4 |
|  | 03BB102 | 34 | 72 | 12 | 3 | 5 | 2 |  | 1 | 2 | 2 | 1 | 2 | 2 | 3 | 2 | 1 | 2 | 3 |
|  | CC-1 | 33 | 59 | 12 | 2 | 1 |  |  | 1 | 2 | 2 | 1 | 2 | 1 | 3 | 2 | 2 | 1 |  |
|  | F837/76 | 31 | 72 | 17 | 3 | 7 | 1 | 1 | 1 | 2 | 2 | 1 | 2 | 1 | 2 | 2 | 1 | 2 |  |
|  | D17 | 29 | 75 | 15 | 3 | 8 | 2 |  | 1 | 2 | 2 | 1 | 2 | 2 | 2 | 2 | 1 | 2 | 4 |
|  | E33L | 24 | 65 | 7 | 2 | 2 | 1 |  | 1 | 2 | 2 | 1 | 2 | 2 | 2 | 2 | 2 | 2 |  |
|  | 97-27 | 22 | 68 | 14 | 2 | 8 | 1 |  | 1 | 2 | 2 | 1 | 2 | 1 | 2 | 2 | 2 | 2 | 1 |
|  | ISSFR-3F | 21 | 83 | 17 | 3 | 4 | 1 |  | 1 | 2 | 2 | 2 | 2 | 2 | 2 | 2 | 2 | 2 |  |
|  | ISSFR-9F | 21 | 82 | 17 | 3 | 4 | 1 |  | 1 | 2 | 2 | 2 | 2 | 2 | 2 | 2 | 2 | 2 |  |
|  | JEM-2 | 21 | 83 | 17 | 3 | 4 | 1 |  | 1 | 2 | 2 | 2 | 2 | 2 | 2 | 2 | 2 | 2 |  |
|  | AH820 | 17 | 66 | 15 | 3 | 7 | 2 |  | 1 | 2 | 2 | 2 | 2 | 2 | 2 | 2 | 2 | 2 | 1 |
|  | 3A | 15 | 75 | 15 | 4 | 2 | 2 |  | 1 | 2 | 3 | 1 | 2 | 2 | 2 | 2 | 2 | 2 | 1 |
|  | S2-8 | 15 | 75 | 15 | 3 | 2 | 2 |  | 1 | 2 | 3 | 2 | 2 | 2 | 2 | 2 | 2 | 2 | 1 |
| *B. cereus biovar anthracis* | CI | 24 | 57 | 14 | 3 | 5 | 1 |  | 1 | 2 | 2 | 1 | 2 | 1 | 2 | 2 | 1 | 2 | 3 |
| *B. thuringiensis*-like | HN001 | 64 | 37 | 17 | 1 | 1 | 1 |  | 1 | 2 | 2 | 1 | 2 | 4 | 3 | 2 | 3 | 2 |  |
|  | CTC | 55 | 38 | 16 | 1 | 1 |  |  | 1 | 2 | 2 | 1 | 2 | 2 | 2 | 2 | 1 | 1 |  |
|  | Bc601 | 53 | 56 | 19 | 2 | 2 |  |  | 1 | 2 | 2 | 1 | 2 | 1 | 3 | 2 | 2 | 2 | 3 |
|  | KNU-07 | 51 | 60 | 27 | 4 | 4 |  |  | 1 | 2 | 2 | 1 | 2 | 2 | 3 | 2 | 2 | 2 | 4 |
|  | FORC_047 | 48 | 31 | 9 | 2 | 2 | 1 |  | 1 | 2 | 2 | 1 | 2 | 2 | 3 | 2 | 3 | 1 |  |
|  | CMCC P0011 | 46 | 26 | 10 | 2 | 4 | 1 |  | 1 | 2 | 2 | 1 | 2 | 4 | 3 | 2 | 2 | 2 | 5 |
|  | CMCC P0021 | 46 | 26 | 10 | 2 | 4 | 1 |  | 1 | 2 | 2 | 1 | 2 | 4 | 3 | 2 | 2 | 2 | 5 |
|  | BM-Bt15426 | 22 | 66 | 16 | 2 | 5 | 1 |  | 1 | 2 | 2 | 2 | 2 | 1 | 2 | 2 | 3 | 2 |  |
| *B. anthracis* | Sterne | 11 | 65 | 11 | 3 | 3 | 2 |  | 1 | 2 | 2 | 1 | 2 | 2 | 2 | 2 | 2 | 1 | 3 |
|  | CDC 684 | 11 | 68 | 12 | 3 | 3 | 2 |  | 1 | 2 | 2 | 1 | 2 | 2 | 2 | 2 | 2 | 1 | 3 |
|  | A0248 | 11 | 65 | 11 | 3 | 3 | 2 |  | 1 | 2 | 2 | 1 | 2 | 2 | 2 | 2 | 2 | 1 | 3 |
|  | H9401 | 11 | 70 | 12 | 3 | 3 | 2 |  | 1 | 2 | 2 | 1 | 1 | 2 | 2 | 2 | 2 | 1 | 3 |
|  | A16 | 11 | 68 | 11 | 3 | 3 | 2 |  | 1 | 2 | 2 | 1 | 2 | 2 | 2 | 2 | 2 | 1 | 3 |
|  | SVA11 | 11 | 63 | 11 | 3 | 3 | 2 |  | 1 | 2 | 2 | 1 | 2 | 2 | 2 | 2 | 2 | 1 | 3 |
| *B. cytotoxicus* | NVH 391-98 | 37 | 1 | 3 | 2 | 10 | 2 |  | 1 | 2 | 1 | 1 | 2 |  |  | 2 |  |  |  |

Additional file 1: Table S3A Correlation pairs for the 102 analyzed *B. cereus s.l.* genomes.

| **Variable 1** | **Variable 2** | **Correlation Coefficient** | **P-value** |
| --- | --- | --- | --- |
| bcr1 | bcr2 | - 0,7493 | <,0001 |
| bcr1 | bcr3 | 0,2834 | 0,0039 |
| bcr1 | bcr5 | - 0,3703 | 0,0001 |
| bcr1 | bcr11 | - 0,3053 | 0,0018 |
| bcr1 | bcr13 | 0,3662 | 0,0002 |
| bcr1 | bcr14 | 0,3419 | 0,0004 |
| bcr1 | bcr15 | 0,3166 | 0,0012 |
| bcr2 | bcr5 | 0,2765 | 0,0049 |
| bcr2 | bcr7 | 0,2074 | 0,0365 |
| bcr2 | bcr10 | 0,1979 | 0,0462 |
| bcr2 | bcr11 | 0,3604 | 0,0002 |
| bcr2 | bcr13 | - 0,4539 | <,0001 |
| bcr2 | bcr15 | - 0,3865 | <,0001 |
| bcr3 | bcr9 | 0,2109 | 0,0334 |
| bcr3 | bcr14 | 0,2483 | 0,0118 |
| bcr3 | bcr17 | 0,2859 | 0,0036 |
| bcr5 | bcr6 | 0,3472 | 0,0004 |
| bcr5 | bcr7 | 0,2769 | 0,0048 |
| bcr5 | bcr11 | 0,223 | 0,0243 |
| bcr5 | bcr14 | - 0,3597 | 0,0002 |
| bcr6 | bcr9 | 0,2315 | 0,0192 |
| bcr6 | bcr11 | 0,2195 | 0,0267 |
| bcr6 | bcr14 | - 0,2978 | 0,0024 |
| bcr7 | bcr10 | 0,2279 | 0,0213 |
| bcr9 | bcr10 | 0,2557 | 0,0095 |
| bcr9 | bcr11 | 0,4619 | <,0001 |
| bcr9 | bcr18 | 0,1982 | 0,0459 |
| bcr10 | bcr12 | - 0,2160 | 0,0293 |
| bcr10 | bcr17 | 0,3821 | <,0001 |
| bcr10 | bcr18 | 0,2112 | 0,0331 |
| bcr11 | bcr14 | - 0,2716 | 0,0057 |
| bcr12 | bcr13 | 0,4069 | <,0001 |
| bcr12 | bcr15 | 0,4200 | <,0001 |
| bcr13 | bcr15 | 0,9609 | <,0001 |
| bcr13 | bcr16 | - 0,2137 | 0,0311 |
| bcr13 | bcr17 | - 0,2531 | 0,0103 |
| bcr14 | bcr16 | 0,2685 | 0,0064 |
| bcr14 | bcr17 | 0,3404 | 0,0005 |
| bcr15 | bcr16 | - 0,2569 | 0,0092 |
| bcr15 | bcr17 | - 0,2954 | 0,0026 |

Additional file 1: Table S3B Correlation pairs for the 102 analyzed *B. cereus s.l.* genomes by species.

|  | Variable 1 | Variable 2 | Correlation Coefficient | P- value (95% confidence interval) |
| --- | --- | --- | --- | --- |
| *B. thuringiensis*  (n=36) | bcr1 | bcr2 | -0,5423 | 0,0006 |
|  | bcr1 | bcr5 | -0,3908 | 0,0185 |
|  | bcr2 | bcr6 | -0,5416 | 0,0007 |
|  | bcr2 | bcr16 | 0,3837 | 0,0209 |
|  | bcr4 | bcr13 | -0,3408 | 0,042 |
|  | bcr5 | bcr8 | 0,3586 | 0,0318 |
|  | bcr6 | bcr9 | 0,3716 | 0,0256 |
|  | bcr6 | bcr13 | 0,4016 | 0,0152 |
|  | bcr9 | bcr10 | 0,5606 | 0,0004 |
|  | bcr9 | bcr11 | 0,5606 | 0,0004 |
|  | bcr10 | bcr17 | 0,4924 | 0,0023 |
|  | bcr11 | bcr14 | -0,3533 | 0,0346 |
|  | bcr12 | bcr16 | 0,4466 | 0,0063 |
|  | bcr16 | bcr17 | -0,3958 | 0,0169 |
| *B. cereus s.s.* (n=42) | bcr1 | bcr2 | -0,7784 | <0,0001 |
|  | bcr1 | bcr5 | -0,3686 | 0,0163 |
|  | bcr1 | bcr6 | -0,3206 | 0,0385 |
|  | bcr1 | bcr11 | -0,5216 | 0,0004 |
|  | bcr1 | bcr14 | 0,7823 | <0,0001 |
|  | bcr2 | bcr5 | 0,4443 | 0,0032 |
|  | bcr2 | bcr10 | 0,102 | 0,0456 |
|  | bcr2 | bcr11 | 0,6032 | <0,0001 |
|  | bcr2 | bcr14 | -0,7035 | <0,0001 |
|  | bcr3 | bcr9 | 0,311 | 0,045 |
|  | bcr5 | bcr6 | 0,547 | 0,0002 |
|  | bcr5 | bcr7 | 0,3473 | 0,0242 |
|  | bcr5 | bcr14 | -0,4347 | 0,004 |
|  | bcr6 | bcr13 | 0,4819 | 0,0012 |
|  | bcr6 | bcr14 | -0,3395 | 0,0278 |
|  | bcr6 | bcr17 | 0,3514 | 0,0225 |
|  | bcr7 | bcr10 | 0,3083 | 0,047 |
|  | bcr7 | bcr14 | -0,334 | 0,0307 |
|  | bcr7 | bcr16 | -0,3284 | 0,0337 |
|  | bcr9 | bcr11 | 0,4676 | 0,0018 |
|  | bcr9 | bcr12 | 0,4878 | 0,001 |
|  | bcr10 | bcr14 | -0,3091 | 0,0464 |
|  | bcr11 | bcr14 | -0,3889 | 0,0109 |
|  | bcr13 | bcr16 | 0,4307 | 0,0044 |
|  | bcr14 | bcr16 | 0,3317 | 0,0319 |
| *B. thuringiensis* -like (n=8) | bcr1 | bcr5 | -0,7906 | 0,0195 |
|  | bcr1 | bcr11 | -0,8733 | 0,0046 |
|  | bcr2 | bcr3 | 0,7611 | 0,0283 |
|  | bcr2 | bcr13 | -0,7827 | 0,0217 |
|  | bcr3 | bcr6 | -0,7159 | 0,0458 |
| *B. mycoides*  (n=4) | bcr1 | bcr2 | 0,9822 | 0,0178 |
|  | bcr1 | bcr6 | 0,9949 | 0,0051 |
|  | bcr1 | bcr16 | 0,9949 | 0,0051 |
|  | bcr1 | bcr18 | 0,9949 | 0,0051 |
|  | bcr2 | bcr6 | 0,9584 | 0,0416 |
|  | bcr2 | bcr16 | 0,9584 | 0,0416 |
|  | bcr2 | bcr18 | 0,9587 | 0,0416 |
|  | bcr6 | bcr16 | 1 | <0,0001 |
|  | bcr6 | bcr18 | 1 | <0,0001 |
|  | bcr12 | bcr13 | 0,9718 | 0,0282 |
|  | bcr12 | bcr14 | -1 | <0,0001 |
|  | bcr12 | bcr15 | 1 | <0,0001 |
|  | bcr12 | bcr17 | -1 | <0,0001 |
|  | bcr13 | bcr14 | -0,9718 | 0,0282 |
|  | bcr13 | bcr15 | 0,9718 | 0,0282 |
|  | bcr13 | bcr17 | -0,9718 | 0,0282 |
|  | bcr14 | bcr15 | -1 | <0,0001 |
|  | bcr14 | bcr17 | 1 | <0,0001 |
|  | bcr15 | bcr17 | -1 | <0,0001 |
|  | bcr16 | bcr18 | 1 | <0,0001 |

Additional file 1: Table S4A Heat map and distribution of group II intron types in the complete genomes of 9 *B. cereus sensu lato* species.


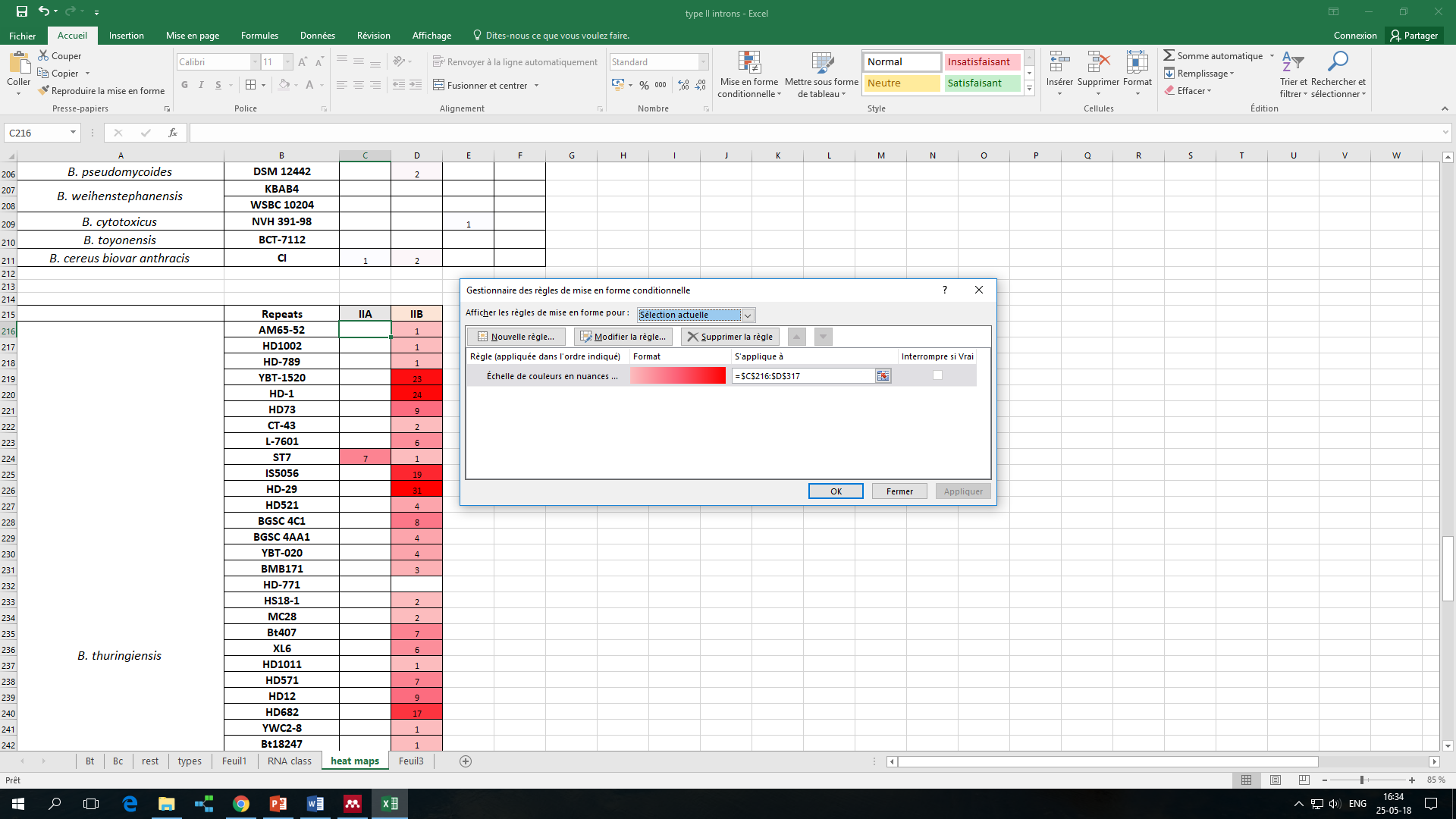


1

31

|  | **Strains** | **IIA** | **IIB** |
| --- | --- | --- | --- |
| *B. thuringiensis* (n=36) | **AM65-52** |  | 1 |
|  | **HD1002** |  | 1 |
|  | **HD-789** |  | 1 |
|  | **YBT-1520** |  | 23 |
|  | **HD-1** |  | 24 |
|  | **HD73** |  | 9 |
|  | **CT-43** |  | 2 |
|  | **L-7601** |  | 6 |
|  | **ST7** | 7 | 1 |
|  | **IS5056** |  | 19 |
|  | **HD-29** |  | 31 |
|  | **HD521** |  | 4 |
|  | **BGSC 4C1** |  | 8 |
|  | **BGSC 4AA1** |  | 4 |
|  | **YBT-020** |  | 4 |
|  | **BMB171** |  | 3 |
|  | **HD-771** |  |  |
|  | **HS18-1** |  | 2 |
|  | **MC28** |  | 2 |
|  | **Bt407** |  | 7 |
|  | **XL6** |  | 6 |
|  | **HD1011** |  | 1 |
|  | **HD571** |  | 7 |
|  | **HD12** |  | 9 |
|  | **HD682** |  | 17 |
|  | **YWC2-8** |  | 1 |
|  | **Bt18247** |  | 1 |
|  | **Bt185** |  | 11 |
|  | **YC-10** |  | 25 |
|  | **YBT-1518** |  | 6 |
|  | **MYBT18246** |  | 1 |
|  | **ATCC10792** |  | 19 |
|  | **SCG04-02** |  | 7 |
|  | **YGD22-03** |  | 2 |
|  | **Bt c25** |  | 1 |
|  | **tolworthi** |  | 22 |
| *B. cereus s.s.* (n=42) | **ATCC 14579** | 1 |  |
|  | **ATCC 10987** | 2 | 8 |
|  | **Q1** |  | 9 |
|  | **B4264** |  |  |
|  | **AH187** | 2 | 13 |
|  | **E33L** | 1 | 5 |
|  | **G9842** |  | 4 |
|  | **AH820** |  | 2 |
|  | **03BB102** |  | 2 |
|  | **F837/76** |  | 2 |
|  | **NC7401** | 2 | 11 |
|  | **FRI-35** |  |  |
|  | **A1** |  |  |
|  | **03BB87** | 2 | 3 |
|  | **D17** | 1 | 5 |
|  | **FM1** |  |  |
|  | **3A** | 2 | 6 |
|  | **G9241** | 1 | 4 |
|  | **ATCC 4342** |  |  |
|  | **03BB108** |  | 6 |
|  | **S2-8** | 2 | 6 |
|  | **FORC_005** |  |  |
|  | **NJ_W** |  |  |
|  | **FORC_013** |  |  |
|  | **FORC_024** | 1 |  |
|  | **AR156** |  |  |
|  | **ISSFR-3F** |  | 1 |
|  | **ISSFR-9F** |  | 1 |
|  | **FT9** | 1 | 4 |
|  | **JEM-2** |  | 1 |
|  | **K8** |  |  |
|  | **M13** |  | 2 |
|  | **C1L** | 2 |  |
|  | **CC-1** |  | 2 |
|  | **D12_2** |  |  |
|  | **FORC021** | 1 | 2 |
|  | **FORC_048** |  |  |
|  | **M3** |  | 7 |
|  | **MLY1** |  | 1 |
|  | **HBL-AI** |  |  |
|  | **Al Hakam** |  | 2 |
|  | **97-27** |  |  |
| *B. thuringiensis*-like (n=8) | **BM-Bt15426** |  |  |
|  | **CTC** |  |  |
|  | **Bc601** |  | 22 |
|  | **KNU-07** |  | 3 |
|  | **CMCC P0011** |  | 1 |
|  | **CMCC P0021** |  | 1 |
|  | **FORC_047** |  |  |
|  | **HN001** |  | 2 |
| *B. anthracis (n=6)* | **Sterne** | 1 | 2 |
|  | **CDC 684** | 1 | 2 |
|  | **A0248** | 1 | 2 |
|  | **H9401** | 1 | 2 |
|  | **A16** | 1 | 2 |
|  | **SVA11** | 1 | 2 |
| *B. mycoides (n=4)* | **ATCC 6462** |  | 1 |
|  | **219298** |  |  |
|  | **BTZ** |  |  |
|  | **Gnyt1** |  | 8 |
| *B. pseudomycoides* | **DSM 12442** |  | 2 |
| *B. weihenstephanensis* (n=2) | **KBAB4** |  |  |
|  | **WSBC 10204** |  |  |
| *B. cytotoxicus* | **NVH 391-98** |  | 1 |
| *B. toyonensis* | **BCT-7112** |  |  |
| *B. cereus biovar anthracis* | **CI** | 1 | 2 |

Additional file 1: Table S4B Heat map and distribution of group II introns in the complete genomes of 9 *B. cereus sensu lato* species.


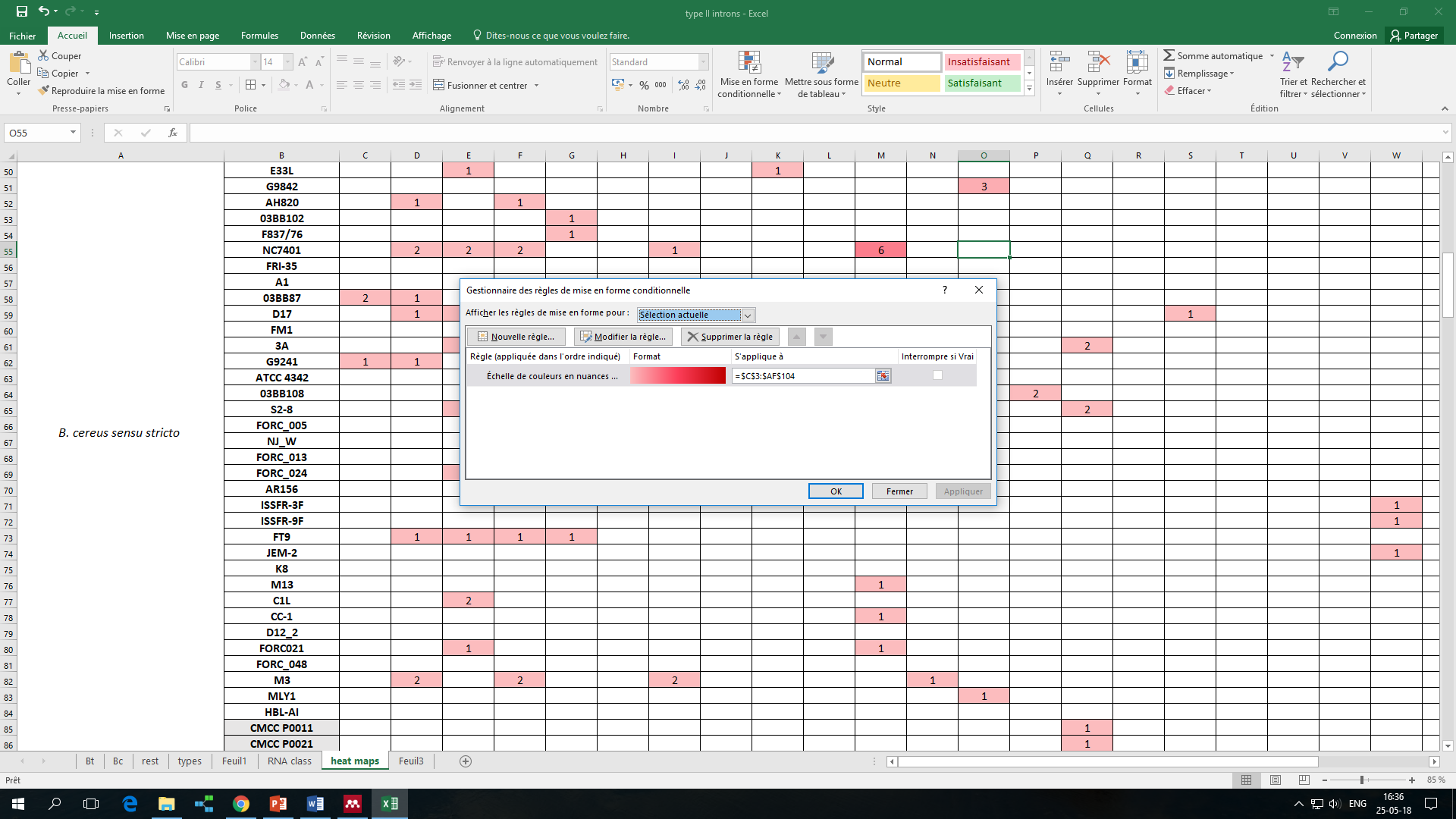


1

19

|  |  |  |  |  |  |  |  |  |  |  |  |  |  |  |  |  |  |  |  |  |  |  |  |  |  |  |  |  | ORF-less | | |
| --- | --- | --- | --- | --- | --- | --- | --- | --- | --- | --- | --- | --- | --- | --- | --- | --- | --- | --- | --- | --- | --- | --- | --- | --- | --- | --- | --- | --- | --- | --- | --- |
|  |  | **B.a.I1** | **B.a.I2** | **B.c.I1** | **B.c.I2** | **B.c.I3** | **B.c.I4** | **B.c.I5** | **B.c.I6** | **B.c.I7a** | **B.c.I8** | **B.c.I10** | **B.c.I11** | **B.c.I12** | **B.c.I14** | **B.c.I15** | **B.c.I16** | **B.c.I17** | **B.c.I18** | **B.my.I1** | **B.ps.I1** | **B.th.I1** | **B.th.I3** | **B.th.I5** | **B.th.I6** | **B.th.I7** | **B.th.I9** | **B.th.I3i** | **B.th.I2** | **B.c.I13** | **B.c.I7b** |
| *B. thuringiensis* | **AM65-52** |  |  |  |  |  |  |  |  |  |  |  |  |  |  |  |  |  |  |  |  |  |  |  |  |  |  | 1 |  |  |  |
|  | **HD1002** |  |  |  |  |  |  |  |  |  |  |  |  |  |  |  |  |  |  |  |  |  |  |  |  |  |  | 1 |  |  |  |
|  | **HD-789** |  |  |  |  |  |  |  |  |  |  |  |  |  |  |  |  |  |  |  |  |  |  |  |  |  |  | 1 |  |  |  |
|  | **YBT-1520** |  |  |  |  |  |  |  |  |  |  |  |  |  |  |  | 2 |  |  |  |  | 6 | 9 | 1 | 2 | 2 |  |  |  |  | 1 |
|  | **HD-1** |  |  |  |  |  |  |  |  |  |  |  |  |  |  |  | 2 |  |  |  |  | 5 | 10 | 1 | 2 | 2 |  |  | 1 |  | 1 |
|  | **HD73** |  |  |  |  |  |  |  |  |  |  |  |  |  |  |  |  |  |  |  |  | 3 | 5 |  |  |  |  |  |  |  | 1 |
|  | **CT-43** |  |  |  |  |  |  |  |  |  |  | 6 |  |  |  |  | 1 |  |  |  |  | 2 | 1 | 1 | 1 | 1 |  |  | 6 |  | 1 |
|  | **L-7601** |  |  |  |  |  |  |  |  |  |  |  |  |  |  |  |  | 1 |  |  |  |  | 4 |  |  |  |  |  |  |  | 1 |
|  | **ST7** |  |  | 7 |  |  |  |  |  |  |  |  |  |  |  |  |  |  |  |  |  |  |  |  |  |  |  |  |  |  | 1 |
|  | **IS5056** |  |  |  |  |  |  |  |  |  |  | 4 |  |  |  |  | 2 |  |  |  |  | 3 | 2 | 1 | 2 | 1 |  |  | 4 |  |  |
|  | **HD-29** |  |  |  |  |  |  |  |  |  |  |  |  |  |  | 1 | 2 | 19 |  |  |  | 2 | 3 | 1 | 2 | 1 |  |  |  |  |  |
|  | **HD521** |  |  |  |  |  |  |  |  |  |  |  |  |  |  |  | 1 | 1 |  |  |  | 1 |  |  | 1 |  |  |  |  |  |  |
|  | **BGSC 4C1** |  |  |  |  |  |  |  |  |  |  |  |  |  |  | 1 | 1 |  |  |  |  | 2 | 2 |  | 1 |  |  |  | 1 |  |  |
|  | **BGSC 4AA1** |  |  |  |  |  |  |  |  |  |  |  |  |  |  | 2 |  |  |  |  |  |  |  |  |  |  |  |  |  |  | 2 |
|  | **YBT-020** |  |  |  |  | 1 |  |  |  | 2 |  |  |  |  |  |  |  |  |  |  |  |  |  |  |  |  | 1 |  |  |  |  |
|  | **BMB171** |  |  |  |  |  |  |  |  |  |  | 1 |  |  |  |  |  |  |  |  |  |  |  |  |  |  |  |  | 1 |  | 1 |
|  | **HD-771** |  |  |  |  |  |  |  |  |  |  |  |  |  |  |  |  |  |  |  |  |  |  |  |  |  |  |  |  |  |  |
|  | **HS18-1** |  |  |  |  |  |  |  |  |  |  |  |  |  |  |  |  |  |  |  |  |  | 2 |  |  |  |  |  |  |  |  |
|  | **MC28** |  |  |  |  |  |  |  |  |  |  |  |  |  |  |  |  |  |  |  |  | 2 |  |  |  |  |  |  |  |  |  |
|  | **Bt407** |  |  |  |  |  |  |  |  |  |  | 2 |  |  |  |  |  |  |  |  |  | 2 | 1 |  |  |  |  |  | 2 |  |  |
|  | **XL6** |  |  |  |  |  |  |  |  |  |  |  |  |  | 3 |  |  |  |  |  |  |  | 1 |  |  |  |  |  |  | 2 |  |
|  | **HD1011** |  |  |  |  |  |  |  |  |  |  |  |  |  |  |  |  |  |  |  |  |  |  |  |  |  |  |  |  |  | 1 |
|  | **HD571** |  |  |  |  | 1 |  |  |  |  |  |  |  |  |  |  |  |  |  |  |  | 5 |  |  |  |  | 1 |  |  |  |  |
|  | **HD12** |  |  |  |  |  |  |  |  | 4 |  | 2 |  |  |  |  |  |  |  |  |  |  |  |  |  |  |  |  | 2 |  | 1 |
|  | **HD682** |  |  |  |  |  |  |  |  |  |  |  |  |  |  |  |  |  |  |  |  | 5 | 12 |  |  |  |  |  |  |  |  |
|  | **YWC2-8** |  |  |  |  |  |  |  |  |  |  |  |  |  |  |  | 1 |  |  |  |  | 3 |  | 1 | 1 | 2 |  |  | 1 |  | 1 |
|  | **Bt18247** |  |  |  |  |  |  |  |  |  |  |  |  |  |  |  |  |  |  |  |  |  |  |  |  |  |  |  |  |  | 1 |
|  | **Bt185** |  |  |  |  |  |  |  |  |  |  |  |  |  |  | 1 | 1 |  |  |  |  | 6 | 1 |  | 1 |  |  |  |  |  | 1 |
|  | **YC-10** |  |  |  |  |  |  |  |  |  |  |  |  |  |  |  | 3 |  |  |  |  |  | 15 | 1 | 2 | 2 |  |  | 1 |  | 1 |
|  | **YBT-1518** |  |  |  |  |  |  |  |  |  |  | 3 |  |  |  |  |  |  |  |  |  |  |  |  |  |  |  |  | 3 |  |  |
|  | **MYBT18246** |  |  |  |  |  |  |  |  |  |  |  |  |  |  |  |  |  |  |  |  | 1 |  |  |  |  |  |  |  |  |  |
|  | **ATCC10792** |  |  |  |  |  |  |  |  |  |  | 5 |  |  |  |  | 1 |  |  |  |  | 3 | 2 |  | 1 |  |  |  | 6 |  | 1 |
|  | **SCG04-02** |  |  |  |  |  |  |  |  |  |  | 2 |  |  |  | 3 |  |  |  |  |  |  |  |  |  |  |  |  | 2 |  |  |
|  | **YGD22-03** |  | 1 |  | 1 |  |  |  |  |  |  |  |  |  |  |  |  |  |  |  |  |  |  |  |  |  |  |  |  |  |  |
|  | **Bt c25** |  |  |  |  |  |  |  |  |  |  |  | 1 |  |  |  |  |  |  |  |  |  |  |  |  |  |  |  |  |  |  |
|  | **tolworthi** |  |  |  |  |  |  |  |  |  |  |  |  |  |  |  |  |  |  |  |  | 2 | 14 | 2 | 3 | 1 |  |  |  |  |  |
| *B. cereus sensu stricto* | **ATCC 14579** |  |  | 1 |  |  |  |  |  |  |  |  |  |  |  |  |  |  |  |  |  |  |  |  |  |  |  |  |  |  |  |
|  | **ATCC 10987** |  | 2 | 2 | 2 | 1 | 1 | 1 |  |  |  |  |  |  |  |  |  |  |  |  |  |  |  |  |  |  | 1 |  |  |  |  |
|  | **Q1** |  | 2 |  | 2 |  |  |  |  |  |  |  | 1 |  |  |  | 1 | 1 | 1 |  |  |  |  |  | 1 |  |  |  |  |  |  |
|  | **B4264** |  |  |  |  |  |  |  |  |  |  |  |  |  |  |  |  |  |  |  |  |  |  |  |  |  |  |  |  |  |  |
|  | **AH187** |  | 2 | 2 | 2 |  |  | 1 |  |  |  | 6 |  |  |  |  |  |  |  |  |  |  |  |  |  |  |  |  | 2 |  |  |
|  | **E33L** |  |  | 1 |  |  |  |  |  | 1 |  |  |  |  |  |  |  |  |  |  |  |  |  |  |  |  |  |  |  |  | 4 |
|  | **G9842** |  |  |  |  |  |  |  |  |  |  |  |  | 3 |  |  |  |  |  |  |  |  |  |  |  |  |  |  |  | 1 |  |
|  | **AH820** |  | 1 |  | 1 |  |  |  |  |  |  |  |  |  |  |  |  |  |  |  |  |  |  |  |  |  |  |  |  |  |  |
|  | **03BB102** |  |  |  |  | 1 |  |  |  |  |  |  |  |  |  |  |  |  |  |  |  |  |  |  |  |  | 1 |  |  |  |  |
|  | **F837/76** |  |  |  |  | 1 |  |  |  |  |  |  |  |  |  |  |  |  |  |  |  |  |  |  |  |  | 1 |  |  |  |  |
|  | **NC7401** |  | 2 | 2 | 2 |  |  | 1 |  |  |  | 6 |  |  |  |  |  |  |  |  |  |  |  |  |  |  |  |  |  |  |  |
|  | **FRI-35** |  |  |  |  |  |  |  |  |  |  |  |  |  |  |  |  |  |  |  |  |  |  |  |  |  |  |  |  |  |  |
|  | **A1** |  |  |  |  |  |  |  |  |  |  |  |  |  |  |  |  |  |  |  |  |  |  |  |  |  |  |  |  |  |  |
|  | **03BB87** | 2 | 1 |  | 1 |  |  |  | 1 |  |  |  |  |  |  |  |  |  |  |  |  |  |  |  |  |  |  |  |  |  |  |
|  | **D17** |  | 1 | 1 |  | 1 |  | 1 |  |  |  |  |  |  |  |  |  | 1 |  |  |  |  |  |  |  |  | 1 |  |  |  |  |
|  | **FM1** |  |  |  |  |  |  |  |  |  |  |  |  |  |  |  |  |  |  |  |  |  |  |  |  |  |  |  |  |  |  |
|  | **3A** |  |  | 2 |  |  |  |  |  |  |  | 2 |  |  |  | 2 |  |  |  |  |  |  |  |  |  |  |  |  | 2 |  |  |
|  | **G9241** | 1 | 1 |  | 2 |  |  |  | 1 |  |  |  |  |  |  |  |  |  |  |  |  |  |  |  |  |  |  |  |  |  |  |
|  | **ATCC 4342** |  |  |  |  |  |  |  |  |  |  |  |  |  |  |  |  |  |  |  |  |  |  |  |  |  |  |  |  |  |  |
|  | **03BB108** |  |  |  |  | 1 |  |  |  |  |  | 1 |  |  | 2 |  |  |  |  |  |  |  |  |  |  |  | 1 |  | 1 |  |  |
|  | **S2-8** |  |  | 2 |  |  |  |  |  |  |  | 2 |  |  |  | 2 |  |  |  |  |  |  |  |  |  |  |  |  | 2 |  |  |
|  | **FORC_005** |  |  |  |  |  |  |  |  |  |  |  |  |  |  |  |  |  |  |  |  |  |  |  |  |  |  |  |  |  |  |
|  | **NJ_W** |  |  |  |  |  |  |  |  |  |  |  |  |  |  |  |  |  |  |  |  |  |  |  |  |  |  |  |  |  |  |
|  | **FORC_013** |  |  |  |  |  |  |  |  |  |  |  |  |  |  |  |  |  |  |  |  |  |  |  |  |  |  |  |  |  |  |
|  | **FORC_024** |  |  | 1 |  |  |  |  |  |  |  |  |  |  |  |  |  |  |  |  |  |  |  |  |  |  |  |  |  |  |  |
|  | **AR156** |  |  |  |  |  |  |  |  |  |  |  |  |  |  |  |  |  |  |  |  |  |  |  |  |  |  |  |  |  |  |
|  | **ISSFR-3F** |  |  |  |  |  |  |  |  |  |  |  |  |  |  |  |  |  |  |  |  | 1 |  |  |  |  |  |  |  |  |  |
|  | **ISSFR-9F** |  |  |  |  |  |  |  |  |  |  |  |  |  |  |  |  |  |  |  |  | 1 |  |  |  |  |  |  |  |  |  |
|  | **FT9** |  | 1 | 1 | 1 | 1 |  |  |  |  |  |  |  |  |  |  |  |  |  |  |  |  |  |  |  |  | 1 |  |  |  |  |
|  | **JEM-2** |  |  |  |  |  |  |  |  |  |  |  |  |  |  |  |  |  |  |  |  | 1 |  |  |  |  |  |  |  |  |  |
|  | **K8** |  |  |  |  |  |  |  |  |  |  |  |  |  |  |  |  |  |  |  |  |  |  |  |  |  |  |  |  |  |  |
|  | **M13** |  |  |  |  |  |  |  |  |  |  | 1 |  |  |  |  |  |  |  |  |  |  |  |  |  |  |  |  | 1 |  |  |
|  | **C1L** |  |  | 2 |  |  |  |  |  |  |  |  |  |  |  |  |  |  |  |  |  |  |  |  |  |  |  |  |  |  |  |
|  | **CC-1** |  |  |  |  |  |  |  |  |  |  | 1 |  |  |  |  |  |  |  |  |  |  |  |  |  |  |  |  | 1 |  |  |
|  | **D12_2** |  |  |  |  |  |  |  |  |  |  |  |  |  |  |  |  |  |  |  |  |  |  |  |  |  |  |  |  |  |  |
|  | **FORC021** |  |  | 1 |  |  |  |  |  |  |  | 1 |  |  |  |  |  |  |  |  |  |  |  |  |  |  |  |  | 1 |  |  |
|  | **FORC_048** |  |  |  |  |  |  |  |  |  |  |  |  |  |  |  |  |  |  |  |  |  |  |  |  |  |  |  |  |  |  |
|  | **M3** |  | 2 |  | 2 |  |  | 2 |  |  |  |  | 1 |  |  |  |  |  |  |  |  |  |  |  |  |  |  |  |  |  |  |
|  | **MLY1** |  |  |  |  |  |  |  |  |  |  |  |  | 1 |  |  |  |  |  |  |  |  |  |  |  |  |  |  |  |  |  |
|  | **HBL-AI** |  |  |  |  |  |  |  |  |  |  |  |  |  |  |  |  |  |  |  |  |  |  |  |  |  |  |  |  |  |  |
|  | **Al Hakam** |  |  |  |  | 1 |  |  |  |  |  |  |  |  |  |  |  |  |  |  |  |  |  |  |  |  | 1 |  |  |  |  |
|  | **97-27** |  |  |  |  |  |  |  |  |  |  |  |  |  |  |  |  |  |  |  |  |  |  |  |  |  |  |  |  |  |  |
| *B. thuringiensis-*like | **CMCC P0011** |  |  |  |  |  |  |  |  |  |  |  |  |  |  | 1 |  |  |  |  |  |  |  |  |  |  |  |  |  |  |  |
|  | **CMCC P0021** |  |  |  |  |  |  |  |  |  |  |  |  |  |  | 1 |  |  |  |  |  |  |  |  |  |  |  |  |  |  |  |
|  | **FORC_047** |  |  |  |  |  |  |  |  |  |  |  |  |  |  |  |  |  |  |  |  |  |  |  |  |  |  |  |  |  |  |
|  | **HN001** |  |  |  |  |  |  |  |  |  |  |  |  |  |  |  |  |  |  |  |  |  |  |  |  |  |  |  |  |  | 2 |
|  | **BM-Bt15426** |  |  |  |  |  |  |  |  |  |  |  |  |  |  |  |  |  |  |  |  |  |  |  |  |  |  |  |  |  |  |
|  | **CTC** |  |  |  |  |  |  |  |  |  |  |  |  |  |  |  |  |  |  |  |  |  |  |  |  |  |  |  |  |  |  |
|  | **Bc601** |  |  |  |  |  |  |  |  |  |  |  |  |  |  |  | 1 |  |  |  |  | 5 | 11 | 1 |  | 2 |  |  | 1 |  | 1 |
|  | **KNU-07** |  |  |  |  |  |  |  |  |  |  |  |  |  |  |  |  |  |  |  |  |  | 3 |  |  |  |  |  |  |  |  |
| *B. anthracis* | **Sterne** | 1 | 1 |  | 1 |  |  |  |  |  |  |  |  |  |  |  |  |  |  |  |  |  |  |  |  |  |  |  |  |  |  |
|  | **CDC 684** | 1 | 1 |  | 1 |  |  |  |  |  |  |  |  |  |  |  |  |  |  |  |  |  |  |  |  |  |  |  |  |  |  |
|  | **A0248** | 1 | 1 |  | 1 |  |  |  |  |  |  |  |  |  |  |  |  |  |  |  |  |  |  |  |  |  |  |  |  |  |  |
|  | **H9401** | 1 | 1 |  | 1 |  |  |  |  |  |  |  |  |  |  |  |  |  |  |  |  |  |  |  |  |  |  |  |  |  |  |
|  | **A16** | 1 | 1 |  | 1 |  |  |  |  |  |  |  |  |  |  |  |  |  |  |  |  |  |  |  |  |  |  |  |  |  |  |
|  | **SVA11** | 1 | 1 |  | 1 |  |  |  |  |  |  |  |  |  |  |  |  |  |  |  |  |  |  |  |  |  |  |  |  |  |  |
| *B. mycoides* | **ATCC 6462** |  |  |  |  |  |  |  |  |  |  |  |  |  |  |  |  |  |  |  |  |  |  |  |  |  |  |  |  |  | 1 |
|  | **219298** |  |  |  |  |  |  |  |  |  |  |  |  |  |  |  |  |  |  |  |  |  |  |  |  |  |  |  |  |  |  |
|  | **BTZ** |  |  |  |  |  |  |  |  |  |  |  |  |  |  |  |  |  |  |  |  |  |  |  |  |  |  |  |  |  |  |
|  | **Gnyt1** |  |  |  |  |  |  |  |  |  |  |  |  |  |  | 1 | 1 |  |  |  |  |  | 2 |  | 2 |  |  |  |  |  | 2 |
| *B. pseudomycoides* | **DSM 12442** |  |  |  |  |  |  |  |  |  |  |  |  |  |  |  |  |  |  | 1 | 1 |  |  |  |  |  |  |  |  |  |  |
| *B. weihenstephanensis* | **KBAB4** |  |  |  |  |  |  |  |  |  |  |  |  |  |  |  |  |  |  |  |  |  |  |  |  |  |  |  |  |  |  |
|  | **WSBC 10204** |  |  |  |  |  |  |  |  |  |  |  |  |  |  |  |  |  |  |  |  |  |  |  |  |  |  |  |  |  |  |
| *B. cytotoxicus* | **NVH 391-98** |  |  |  |  |  |  |  |  |  | 1 |  |  |  |  |  |  |  |  |  |  |  |  |  |  |  |  |  |  |  |  |
| *B. toyonensis* | **BCT-7112** |  |  |  |  |  |  |  |  |  |  |  |  |  |  |  |  |  |  |  |  |  |  |  |  |  |  |  |  |  |  |
| *B. cereus biovar anthracis* | **CI** | 1 | 1 |  | 1 |  |  |  |  |  |  |  |  |  |  |  |  |  |  |  |  |  |  |  |  |  |  |  |  |  |  |
